# Identification of Evolutionarily Conserved Exons as Regulated Targets for the Splicing Activator Tra2β in Development

**DOI:** 10.1371/journal.pgen.1002390

**Published:** 2011-12-15

**Authors:** Sushma Grellscheid, Caroline Dalgliesh, Markus Storbeck, Andrew Best, Yilei Liu, Miriam Jakubik, Ylva Mende, Ingrid Ehrmann, Tomaz Curk, Kristina Rossbach, Cyril F. Bourgeois, James Stévenin, David Grellscheid, Michael S. Jackson, Brunhilde Wirth, David J. Elliott

**Affiliations:** 1 Institute of Genetic Medicine, Newcastle University, Newcastle, United Kingdom; 2 Institute of Human Genetics, University of Cologne, Cologne, Germany; 3 Institute of Genetics, University of Cologne, Cologne, Germany; 4 Center for Molecular Medicine, University of Cologne, Cologne, Germany; 5 University of Ljubljana, Faculty of Computer and Information Science, Ljubljana, Slovenia; 6 Department of Functional Genomics and Cancer, Institut de Génétique et de Biologie Moléculaire et Cellulaire (IGBMC), INSERM U 964, CNRS UMR 7104, Université de Strasbourg, Illkirch, France; 7 Institute for Particle Physics Phenomenology, Durham University, Durham, United Kingdom; Medical Research Council Human Genetics Unit, United Kingdom

## Abstract

Alternative splicing amplifies the information content of the genome, creating multiple mRNA isoforms from single genes. The evolutionarily conserved splicing activator Tra2β (Sfrs10) is essential for mouse embryogenesis and implicated in spermatogenesis. Here we find that Tra2β is up-regulated as the mitotic stem cell containing population of male germ cells differentiate into meiotic and post-meiotic cells. Using CLIP coupled to deep sequencing, we found that Tra2β binds a high frequency of exons and identified specific G/A rich motifs as frequent targets. Significantly, for the first time we have analysed the splicing effect of *Sfrs10* depletion *in vivo* by generating a conditional neuronal-specific *Sfrs10* knock-out mouse (*Sfrs10^fl/fl^*; *Nestin-Cre^tg/+^*). This mouse has defects in brain development and allowed correlation of genuine physiologically Tra2β regulated exons. These belonged to a novel class which were longer than average size and importantly needed multiple cooperative Tra2β binding sites for efficient splicing activation, thus explaining the observed splicing defects in the knockout mice. Regulated exons included a cassette exon which produces a meiotic isoform of the Nasp histone chaperone that helps monitor DNA double-strand breaks. We also found a previously uncharacterised poison exon identifying a new pathway of feedback control between vertebrate Tra2 proteins. Both *Nasp-T* and the *Tra2a* poison exon are evolutionarily conserved, suggesting they might control fundamental developmental processes. Tra2β protein isoforms lacking the RRM were able to activate specific target exons indicating an additional functional role as a splicing co-activator. Significantly the N-terminal RS1 domain conserved between flies and humans was essential for the splicing activator function of Tra2β. Versions of Tra2β lacking this N-terminal RS1 domain potently repressed the same target exons activated by full-length Tra2β protein.

## Author Summary

Alternative splicing amplifies the informational content of the genome, making multiple mRNA isoforms from single genes. Tra2 proteins bind and activate alternative exons, and in mice Tra2β is essential for embryonic development through unknown target RNAs. Here we report the first target exons that are physiologically regulated by Tra2β in developing mice. Normal activation of these regulated exons depends on multiple Tra2β binding sites, and significant mis-regulation of these exons is observed during mouse development when Tra2β is removed. As expected, Tra2β activates splicing of some target exons through direct RNA binding via its RNA Recognition Motif. Surprisingly, for some exons Tra2β can also activate splicing independent of direct RNA binding through two domains enriched in arginine and serine residues (called RS domains). The N-terminal RS1 domain of Tra2β is absolutely essential for splicing activation of physiological target exons, explaining why this domain is conserved between vertebrates and invertebrates. Surprisingly, Tra2β proteins without RS1 operate as splicing repressors, suggesting the possibility that endogenous Tra2β protein isoforms may differentially regulate the same target exons.

## Introduction

Almost all transcripts from genes encoding multiple exons are alternatively spliced, and correct patterns of alternative splicing are important for health and normal development [1], [2], [3]. Alternative splicing introduces new coding information into mRNAs, thereby increasing genome capacity to encode an expanded number of mRNAs and proteins from a finite number of genes [3]. Poison exons which introduce premature stop codons can also be alternatively spliced to target mRNAs for degradation through Nonsense Mediated Decay (NMD) [4], [5], [6], [7], .

Alternative splice events are controlled in part by *trans-* acting RNA binding proteins which help establish patterns of alternative splicing through deciphering a splicing code embedded within the pre-mRNA sequence [9], [10], [11]. Tra2 proteins bind directly to target exons thereby activating splicing inclusion [12], and have a modular organisation comprising a single central RNA recognition motif (RRM) which binds to target RNA sequences, flanked by arginine-serine rich (RS1 and RS2) domains [13], [14]. The N-terminal Tra2 RS1 domain is longer and contains more RS dipeptides than RS2. The reason for this unique modular organisation is unknown, but is conserved in vertebrate and invertebrate Tra2 proteins and different from the classical SR super-family which have a single C-terminal RS domain [15]. Also unlike classical SR proteins, Tra2 proteins do not restore splicing activity to S100 extracts [12].

A single Tra2 protein is conserved in fruit flies, where it is essential for spermatogenesis and sex determination [16]. There are two mammalian Tra2 proteins called Tra2α (encoded by the *Tra2a* gene on mouse chromosome 6) and Tra2β (encoded by the *Sfrs10* gene on mouse chromosome 16) which share 63% amino acid identity and similar RNA binding specificities [12]. NMR analyses have recently shown that the optimal core RNA target sequence for binding full length Tra2β protein is an AGAA motif, with each of the nucleotide residues being specifically recognized by the Tra2β RRM [17], [18].

A key priority to understand the biological functions of Tra2β is to identify target RNAs which are functionally regulated within animal cells, and associated pathways of gene activity. Mice with ubiquitous deficiency of the *Sfrs10* gene die at around 7.5 to 8.5 days of gestation [19]. Splicing of some Tra2β candidate target exons have been investigated using minigenes, but recently a well known regulated splice target exon (*SMN2* exon 7) was found to have the same splicing pattern within wild type mice and *Smn^−/−^; SMN2^tg/tg^*; *Sfrs10^−/−^* mouse cells which do not express Tra2β protein [19]. These data suggest Tra2β is not the key protein regulating physiological inclusion of *SMN2* exon 7 within animal cells.

**Figure 1:**
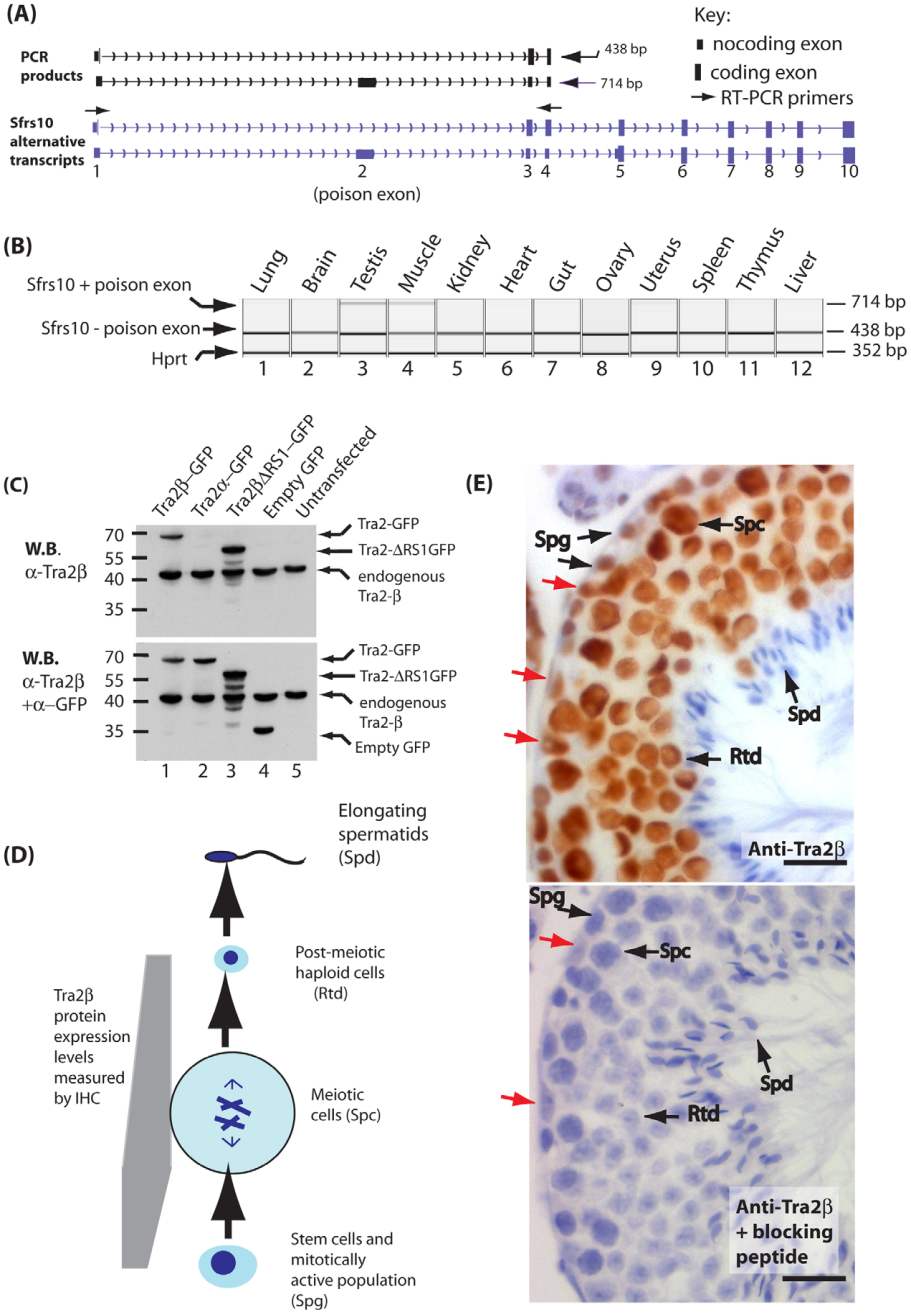
Tra2β is a nuclear protein highly expressed in mouse germ cells. (A) Diagram showing *in silico* PCR of the mouse *Sfrs10* mRNA redrawn from the UCSC mouse genome browser [69]. Two different RT-PCR products are amplified using primers in exons 1 and 4. The smaller product (438 nucleotides) represents the amplified product when exon 1 is directly spliced to exon 3 and then exon 3 to exon 4 (upper *Sfrs10* mRNA isoform). The larger product (714 nucleotides) represents when the poison exon 2 is spliced resulting in the non-translated isoform Tra2β4 (lower *Sfrs10* mRNA isoform). (B) Capillary gel electrophoresis image showing levels levels of *Sfrs10* mRNA assayed by multiplex RT-PCR using RNA purified from adult mouse tissues. Primers were used for amplification complementary to exons 1 and 4 as described in (A) above. Within a multiplex RT-PCR, primers were included to detect *Hprt* as a parallel loading control to ensure equivalent amounts of RNA were used in each lane. (C) Immunoblotting experiment to confirm the specificity of the polyclonal antisera used for immunohistochemistry. HEK293 cells were transfected with plasmids expressing the indicated proteins. Proteins were then isolated and analysed by SDS-PAGE and Western blotting. The same blot was probed sequentially with an affinity purified antisera ab31353 raised against Tra2β (top panel) and then with a polyclonal specific for GFP to detect overall expression of each of the fusion proteins (lower panel). The ab31353 α-Tra2β antisera detected a single band in HEK293 cells corresponding to endogenous Tra2β protein, and in transfected cells additionally detected the Tra2β-GFP fusion protein and Tra2βΔRS1-GFP. No cross reaction with Tra2α-GFP was observed, indicating that this purified antisera is highly specific to Tra2β. (D) Flow chart summarising major developmental stages in male germ cell development. (E) Tra2β is a nuclear protein expressed during and after meiosis. Paraffin embedded adult mouse testis sections were stained with an affinity purified antibody raised against Tra2β (brown staining), and counterstained with haematoxylin (blue). Abbreviations: Spg –spermatogonia (mitotically active population which includes stem cells); Spc –spermatocyte (meiotic cells); Rtd –round spermatid (post-meiotic haploid cell); Spd –elongating spermatid (differentiating haploid cell with condensed nuclei). The scale bar is equivalent to 20 µm. The red arrows indicate Sertoli Cells. Based on these immunohistochemistry results, the Tra2β protein expression levels during mouse germ cell development are summarised also on the flow chart in part (D).

The *Sfrs10* gene itself is alternatively spliced to five mRNA isoforms encoding at least 2 protein isoforms [20], [21], [22]. The major isoform encodes full length Tra2β protein. Full length Tra2β protein regulates its own levels through activating splicing inclusion of a poison exon (exon 2) into a second mRNA isoform, preventing protein translation (Figure 1A) [22]. A third mRNA isoform encodes just the C-terminus of the protein (containing the RRM, glycine linker and the RS2 domain) giving rise to the protein isoform Tra2beta-3 or Tra2βΔRS1 [20], [21], [22]. No distinct function has been assigned to the Tra2βΔRS1 isoform compared to full length Tra2β [17], although this isoform is conserved in invertebrates so likely important. Tra2βΔRS1 expression is tissue specific in both flies and mammals, and is up-regulated by expression of Clk kinases and neural stimulation [20], [21], [22], [23].

Male germ cell development is one of the few developmental pathways to continue into the adult. In the fly testis, Tra2 regulates splicing of *Exuperentia* and *Att* pre-mRNAs in male germ cells, as well as its own alternative splicing pathway [24], [25]. Tra2β has been implicated in mammalian spermatogenesis through interaction with RBMY protein which is genetically deleted in some infertile men [26], [27], and regulates the splicing of the human testis-specific HIPK3-T exon through a switch-like mechanism [28], [29]. Given its important role in *Drosophila* spermatogenesis and established interactions with proteins implicated in human male fertility we predicted that Tra2β-regulated alternative splicing events would control fundamental pathways in mammalian male germ cell development. We have tested this prediction here using a transcriptome-wide approach.

## Results

### Tra2β is ubiquitously expressed but up-regulated at the onset of meiosis in male germ cells

We analysed the expression of *Sfrs10* mRNA in different adult mouse (*Mus musculus*) tissues by RT-PCR using primers in exons 1 and 4. An RT-PCR product derived from *Sfrs10* mRNA in which exons 1 and 3 were directly spliced (skipping poison exon 2) was seen in every tissue indicating the *Sfrs10* gene is ubiquitously expressed (Figure 1A and 1B). A larger *Sfrs10* RT-PCR product made from mRNAs including poison exon 2 was detected at high levels in just two tissues, testis and muscle, indicating that expression of Tra2β is particularly tightly controlled in these tissues [22]. Similar levels of expression of *Hprt* mRNA were observed in each tissue by multiplex RT-PCR.

A polyclonal antiserum raised to Tra2β protein identified a single endogenous protein of around 40 KDa in both transfected and untransfected HEK293 cells corresponding in size to endogenous Tra2β (Figure 1C). A Tra2β-GFP fusion protein was additionally detected within transfected cells, but no cross-reaction was detected with a Tra2α-GFP fusion indicating high specificity of the antiserum. We were also able to detect a GFP-fusion protein containing Tra2βΔRS1, but not endogenous Tra2βΔRS1 protein suggesting that this particular isoform is expressed at low levels in these cells. Further probing of the same filter indicated that all the GFP fusion proteins were expressed at similar levels (Figure 1C, lower panel).

We used indirect immunohistochemistry to determine the cell type distribution of full length Tra2β in the adult testis (Figure 1D and 1E). Tra2β was detected as a nuclear protein (Figure 1E upper panel), and all staining was prevented by pre-incubation of the antisera with the immunising peptide (Figure 1E lower panel). Tra2β was most highly expressed during mouse male germ cell development at the meiotic stage in spermatocytes (abbreviated Spc), and afterwards in round spermatids (abbreviated Rtd). Less intense Tra2β staining was detected within spermatogonia which contain the mitotically active stem cell population. No immunostaining was detected in elongating spermatids (abbreviated Spd). This regulated expression pattern predicts that Tra2β might play a role in regulating meiotic and post-meiotic exon inclusion during male germ cell development. Outside the germline, Tra2β protein expression was detected in Sertoli cells (indicated by red arrows on Figure 1E).

### Tra2β primarily binds AGAA-rich target sequences in mouse germ cells

To identify endogenous cellular RNA targets for Tra2β we carried out high throughput sequencing cross linking immunoprecipitation (HITS-CLIP) [30]. Adult mouse testis cells were used according to published procedures (see methods for details) to retrieve an average tag length of 40 nucleotides. These recovered CLIP tags correspond to specific RNA sequences bound and subsequently cross-linked to endogenous Tra2β protein within the testis.

**Figure 2:**
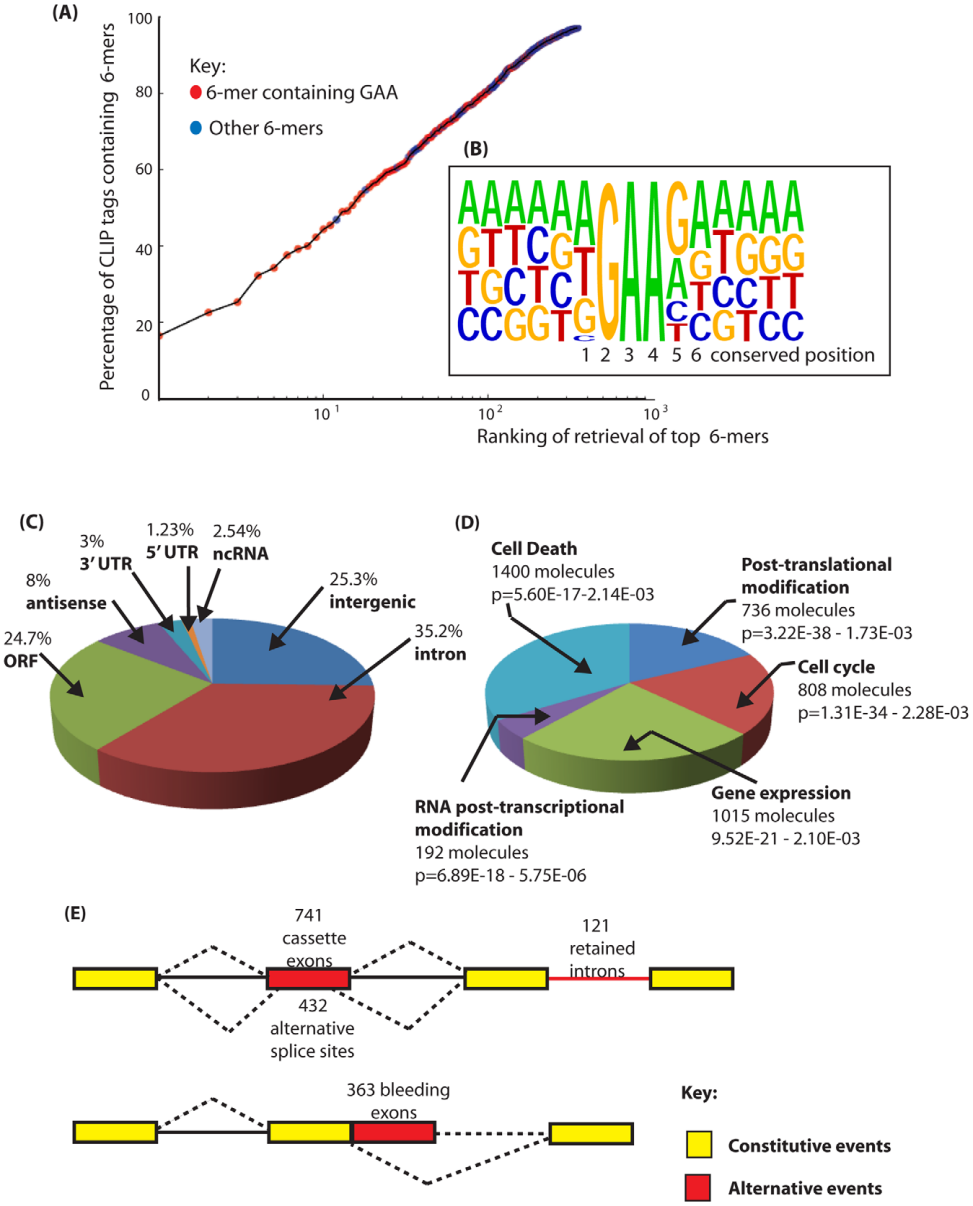
Identification of binding sites for Tra2β in the mouse transcriptome. (A) Nucleotide sequences enriched in the Tra2β CLIP tags are enriched in the core motif GAA. The percentage of CLIP tags was plotted against the order of retrieval of individual 6-mers on a logarithmic scale to identify the most frequently occurring 6-mer sequences within the CLIP tags. CLIP tag sequences which contain GAA are indicated in red. All other CLIP tags are shown in blue. (B) Consensus binding site for Tra2β derived from alignment of full length CLIP tags. The consensus was constructed by anchoring CLIP tags around GAA and then performing an alignment. The positions 1–6 which are particularly conserved are shown underneath and discussed in the main text. (C) Pie chart showing percentage of retrieved CLIP tags mapping to different inter- and intragenic locations within the mouse transcriptome. (D) Summary of the top 5 molecular and cellular functions for Tra2β determined by Ingenuity Pathway Analysis. (E) Distribution of Tra2β binding sites relative to the different classes of alternative events annotated on the mouse genome. Alternative events are shown in red, and the constitutive events as yellow boxes (exons) or black lines (introns). Alternative events are annotated according to the UCSC genome browser track Alternative Events (URL: http://genome.ucsc.edu/cgi-bin/hgTrackUi?g=knownAlt&hgsid=212031267).

To identify frequent physiological Tra2β binding sites in mouse testis we searched for frequently occurring 6-mers in the retrieved CLIP tags, and normalised these to their background occurrence in the mouse genome and transcriptome using custom-written Python scripts (Table S1 and Table S2). Each of the most frequently recovered 6-mers was significantly enriched in the CLIP dataset compared to their representation in the mouse genome or mouse testis transcriptome. Strikingly, purine-rich sequences were preferentially recovered in our CLIP tags. In fact, 14 hexamers out of the top 30 recovered genome corrected hexamers in Table S1 have only purine residues, and 13 have only one pyrimidine. More specifically and consistent with the known RNA binding site for Tra2β [17], [18], GAA-containing sequences were frequently observed. The distribution of GAA-containing 6-mers in the overall population of CLIP tags was visualised by plotting the genomic ranking of 6-mer recovery (X axis) against their representation in the CLIP population (Y axis) (Figure 2A: GAA-containing 6-mers are shown in red, with all other 6-mer sequences in blue). Of the 30 most frequently recovered 6-mers, 27 had a core GAA motif and the other 3 an AGA motif. The most frequent 6-mer (the AGAAGA motif, 10° on the X axis of Figure 2A -equivalent to 1) was found in almost 20% of the recovered CLIP tags. The ten most frequently recovered 6-mers were found in more than 40% of the CLIP tags.

Next we aligned full length CLIP tags to generate a transcriptome-wide consensus sequence. We anchored this line-up between CLIP tags using the trinucleotide GAA from the core binding motif which is essential for efficient RNA protein interactions [17] (Figure 2B). Within this consensus alignment, an A residue followed by a T residue (and less frequently a G residue) was usually found upstream of the GAA motif (position 1 in Figure 2B), consistent with reported *in vitro* RNA-protein binding data between the RRM of Tra2β and synthetic oligonucleotides [17]. Furthermore, a G residue (and less frequently an A residue) was preferentially selected at the position downstream of the GAA motif (position 5), and an A at the next nucleotide position downstream (position 6). This results in an extended AGAAGA consensus, in agreement with the sequence of the 3 top hexamers. Interestingly, when only a GAA triplet but not an AGAA core is present within a CLIP tag, 89% of the tags have a G residue immediately downstream (GAAG), consistent with the important contribution of the G5 residue for efficient binding of Tra2β to its natural RNA targets. No further strong sequence bias was noticed in the sequences upstream and downstream of the AGAAGA hexamer. A similar consensus was obtained previously for SRSF1 protein [31]. However since SRSF1 has 2 RRMs with different RNA binding capacities and only one RS domain, it is most likely that its global specificity of RNA recognition and binding are broader than that for Tra2β and also depends on other ESEs within its individual target exons.

### Tra2β binds a high frequency of exonic sequences

To identify specific endogenous target transcripts CLIP tags were mapped onto the mouse genome sequence (a full bed file of Tra2β CLIP tags is provided as Dataset S1) [32]. Overall, the distribution of Tra2β CLIP tags was predominantly intragenic: Around 69% of Tra2β binding sites were located within protein coding genes, even though genes contribute just 25% of the genome (Figure 2C). Network analyses indicated the main functional properties associated withTra2β target transcripts were post-translational modification, the cell cycle, gene expression, RNA post-transcriptional modification and cell death (Figure 2D). Top physiological systems associated with Tra2β target transcripts included reproductive system and nervous system development, and there was significant enrichment of signalling pathways in the top detected pathways (Table S3). Most intragenic CLIP tags mapped to transcripts in the sense orientation, but 7.5% of retrieved CLIP tags were antisense to known annotated genes.

Only 1.3% of the mouse genome encodes exons (5′ UTR, ORF and 3′ UTR, based on mm9 annotation version ensembl59). For Tra2β some 29% of Tra2β CLIP tags mapped within exons of protein coding genes (Figure 2C) which indicates the presence of numerous Tra2β-specific target exons. Similar CLIP-based transcriptome-wide analyses found that the SR protein SRSF1 also frequently binds to exonic sequences, while Nova and PTB target sites are mainly intronic in distribution [30], [31], [33].

Non-exonic Tra2β binding sites were found within deep intronic regions, within locations annotated as intergenic and within noncoding RNAs (ncRNAs) [34]. Within ncRNAs Tra2β binding sites were found within the small subunit rRNA (also identified as a binding site for SRSF1 [31]) and 7SK RNA. There were also Tra2β binding sites within the ncRNA *Malat1* which is known to be localised in nuclear splicing speckles enriched in pre-mRNA splicing components (*Malat1* is also bound by SRSF1 [31]), and within microRNAs. These identified targets suggest that Tra2β might in fact be a somewhat multifunctional post-transcriptional regulator. Similarly diverse classes of target RNA (including both coding and ncRNAs) have been identified for a number of other RNA binding proteins by HITS-CLIP [30], [31], [33], [35], [36].

### Analysis of endogenous target exons indicate that isoforms of Tra2β can activate, co-activate, and repress exon inclusion

**Figure 3:**
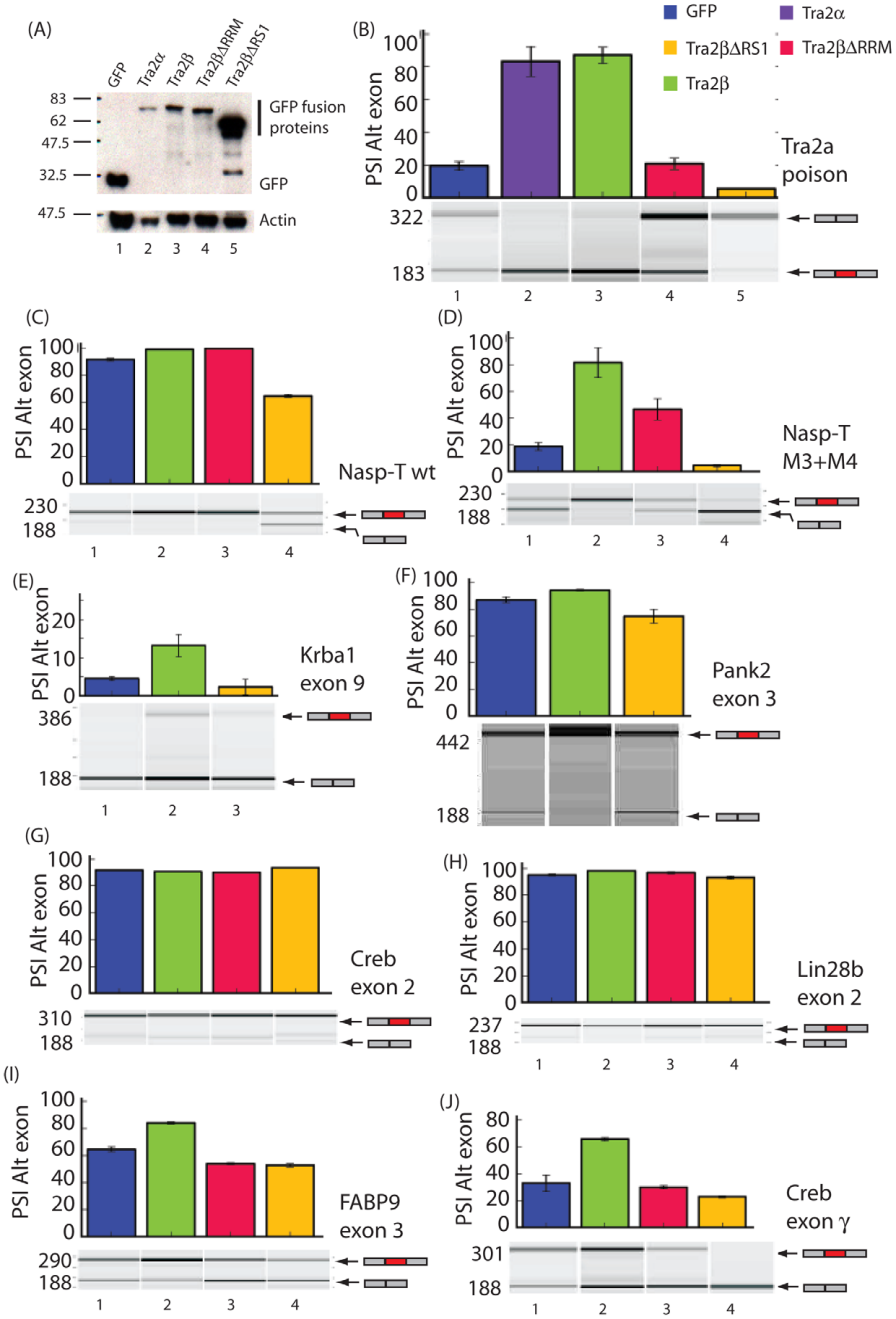
Different protein isoforms of Tra2β can act as specific splicing activators, co-activators, and repressors of a target exons identified by HITS-CLIP. (A) Efficient protein expression levels of different GFP fusion proteins used in these experiments (upper panel). Levels of actin were measured in parallel (lower panel). (B)–(J). Upper panels: Bar charts showing percentage splicing inclusion (PSI) of a panel of exons identified through HITS-CLIP in response to GFP and Tra2β-GFP fusion proteins. All data used to make the bar charts was from at least 3 biological replicates, and the error bars are shown as standard errors. Lower panels: Representative capillary gel electrophoresis image from each RT-PCR analysis. Probability (p) values were calculated using an independent two-sample T-test between the PSI levels for cells co-transfected with GFP and each of the different Tra2β-GFP constructs (* p≤0.05, **p≤0.01).

Tra2β bound to both constitutive and alternative exons and also to each different class of alternative events annotated on the mouse genome browser at UCSC. In particular, Tra2β binding sites mapped preferentially to cassette exons (this is also the most frequent class of alternative splicing event in metazoans [37]) (Figure 2E). To test for splicing regulation of these identified target exons by Tra2β, a panel of seven cassette exons with high numbers of mapped CLIP tags, together with flanking intronic sequences, were cloned into an exon trap vector (see Materials and Methods). The resulting minigenes were then transfected into HEK293 cells with expression constructs encoding either GFP, Tra2β-GFP, or GFP-tagged Tra2β deletion variants. Western blots indicated each of the GFP-fusion proteins were efficiently expressed in HEK293 cells (Figure 3A), although the fusion protein without the RS1 domain was expressed at higher levels.

Splicing patterns of pre-mRNAs were analysed using RT-PCR. We observed particularly strong splicing activation of a poison exon in the *Tra2a* gene in response to co-expression of Tra2β-GFP (Figure 3B). Ectopic expression of both Tra2α and Tra2β were equally able to activate splicing of the *Tra2a* poison exon indicating that these two proteins are functionally equivalent in this assay (Figure 3B, lanes 2 and 3). No splicing activation of the *Tra2a* poison exon was observed with either Tra2βΔRRM-GFP or GFP alone, indicating a requirement for RRM-dependent binding by full length Tra2β proteins for splicing activation (Figure 3B, lanes 1 and 4).

Full length Tra2β also mediated statistically significant splicing activation of a cassette exon annotated *Nasp-T* in the *Nasp* gene. Surprisingly, equally strong and highly statistically significant *Nasp-T* exon splicing activation was also observed in response to ectopic expression of Tra2βΔRRM-GFP protein (Figure 3C, lanes 2 and 3). Because of the high levels of splicing inclusion observed for the wild type *Nasp-T* exon at endogenous cellular concentrations of Tra2β (Figure 3C), we also repeated these experiments using a mutated exon which is less efficiently spliced (mutant M3+M4 –see below) and again observed significant splicing activation by Tra2βΔRRM-GFP protein (Figure 3D –in this case the effect of Tra2βΔRRM-GFP is clearer because of the lower levels of splicing inclusion of this mutated exon at endogenous cellular Tra2β protein concentrations). Together these data indicate that for some exons including Nasp-T, Tra2β can activate splicing through RRM independent interactions as well as being a direct splicing activator as previously described.

The *Sfrs10* locus encodes a second endogenous protein isoform called Tra2βΔRS1 [20], [21], [22] which lacks the RS1 domain. Surprisingly, after co-expression of a Tra2β-GFPΔRS1 protein isoform we observed significant splicing repression of both the *Tra2a* poison exon and *Nasp-T* exon (Figure 3B–3D) indicating that this protein isoform behaves as a potent splicing repressor, and of the same target exons recognised by full length Tra2β protein.

Two further exons, *Creb* exon 2 and *Lin28b* exon 2, did not detectably respond to ectopic expression of full length Tra2β or any of its derivatives (Figure 3G and 3H) and were already included at high levels in the absence of ectopically expressed Tra2β protein. No strong splicing repression of *Creb* exon 2 and *Lin28b* exon 2 was observed on co-expression of Tra2β-GFPΔRS1. Full length Tra2β weakly but significantly activated splicing of two other target exons, *Krba1* exon 9 and *Pank2* exon 3 (Figure 3E and 3F) and splicing of these exons was also not significantly repressed by Tra2β-GFPΔRS1 (compare lanes 1 and 3: notice slight repression which was not statistically significant). We also looked at two other exons which are spliced in the testis and which we independently characterised as being regulated by Tra2β. Minigene experiments indicated both the *Crebγ* and *Fabp9* exons [38], [39] were moderately activated by Tra2β, and were also co-ordinately moderately repressed by the Tra2βΔRS1 isoform (Figure 3I and 3J, lanes 1 and 4). Taken together these data are consistent with full length Tra2β protein activating specific target exons, and the Tra2βΔRS1 protein isoform specifically repressing exons which are at least moderately to strongly activated by full length Tra2β, but not acting as a general repressor of cellular splicing.

### Tra2β directly binds to target transcripts identified by CLIP, and binding efficiency correlates with splicing activity

We carried out further *in silico* and molecular analyses to correlate Tra2β binding with the observed patterns of exon regulation. We firstly looked for the occurrence of over-represented transcriptome-wide enriched 6-mer sequences (k-mers) [40] to identify putative Tra2β binding sites in the analysed target exons *in silico* (Figure S1). Both the *Nasp-T* and *Tra2a* poison exon had a high predicted content of 6-mers corresponding to putative Tra2β binding sites and consistent with their strong Tra2β regulation observed *in vitro*.

**Figure 4:**
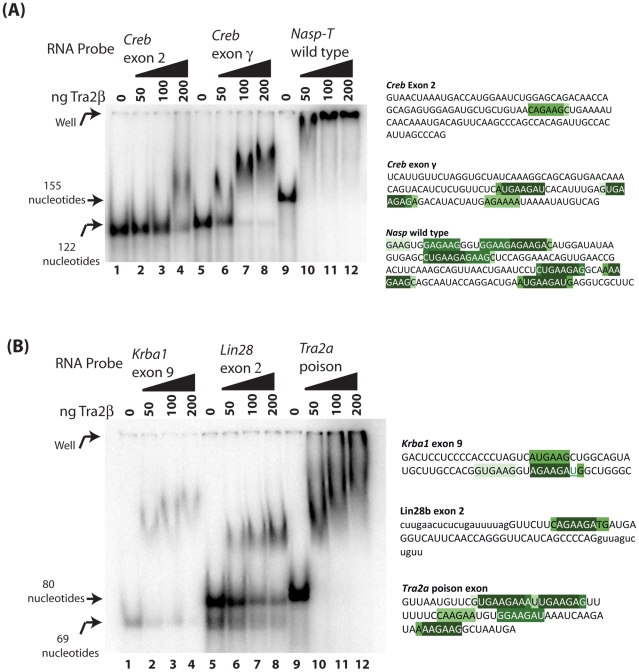
Tra2β CLIP targets bind to full length Tra2β protein. (A) EMSAs of *Creb* exon 2, *Creb* exon γ and the wild type *Nasp-T* exon. (B) EMSAs of *Krba1* exon 9, *Lin28* exon 2 and the *Tra2a* poison exon. Electrophoretic Mobility Shift Assays (EMSAs) were carried out with full length Tra2β protein and short radioactive RNA probes from pre-mRNAs identified by CLIP and which contained predicted Tra2β protein binding sites from the transcriptome-wide 6-mer analysis. The RNA probes are shown to the right of the gel panels, and the sequences are highlighted for different categories of 6-mers as in Table S1. Exon sequences are shown in upper case, and any flanking intron sequence in lower case (the *Lin28b* exon is very short).

We then directly measured Tra2β binding affinities using Electromobility Shift Assays (EMSAs) (Figure 4: the positions of predicted binding sites within the RNA probes are shaded as in Table S1. Notice the dark green corresponds to the top 5 most frequently recovered 6-mers, and lighter shades of green correspond to less frequently recovered 6-mers). Both *Nasp-T* and *Tra2a* poison exon probes were very efficiently shifted by even very low concentrations of Tra2β protein (the *Nasp-T* probe was shifted into the well by only 50 ng of added Tra2β protein indicating formation of very large Tra2β protein-RNA complexes, and increasing molecular weight *Tra2a* RNA-protein complexes were observed with increasing concentrations of full length Tra2β protein).

A series of increased molecular weight complexes also formed on the *Crebγ* exon RNA probe (corresponding exon regulated *in cellulo* by Tra2β) and on the *Krba1* RNA probe (weakly responsive *in cellulo* to Tra2β splicing activation). A single higher molecular weight complex formed on the *Lin28* probe (exon splicing not activated *in vitro* by Tra2β, and contains a single predicted Tra2β binding site). Much less efficient binding was observed for the non Tra2β-responsive *Creb* exon 2 (which formed a single molecular weight complex only with 200 ng added Tra2β protein, compared with 50 ng for the *Crebγ* probe).

### The *Tra2a* poison and *Nasp-T* cassette exons are phylogenetically conserved and show high levels of splicing inclusion in mouse testis

**Figure 5:**
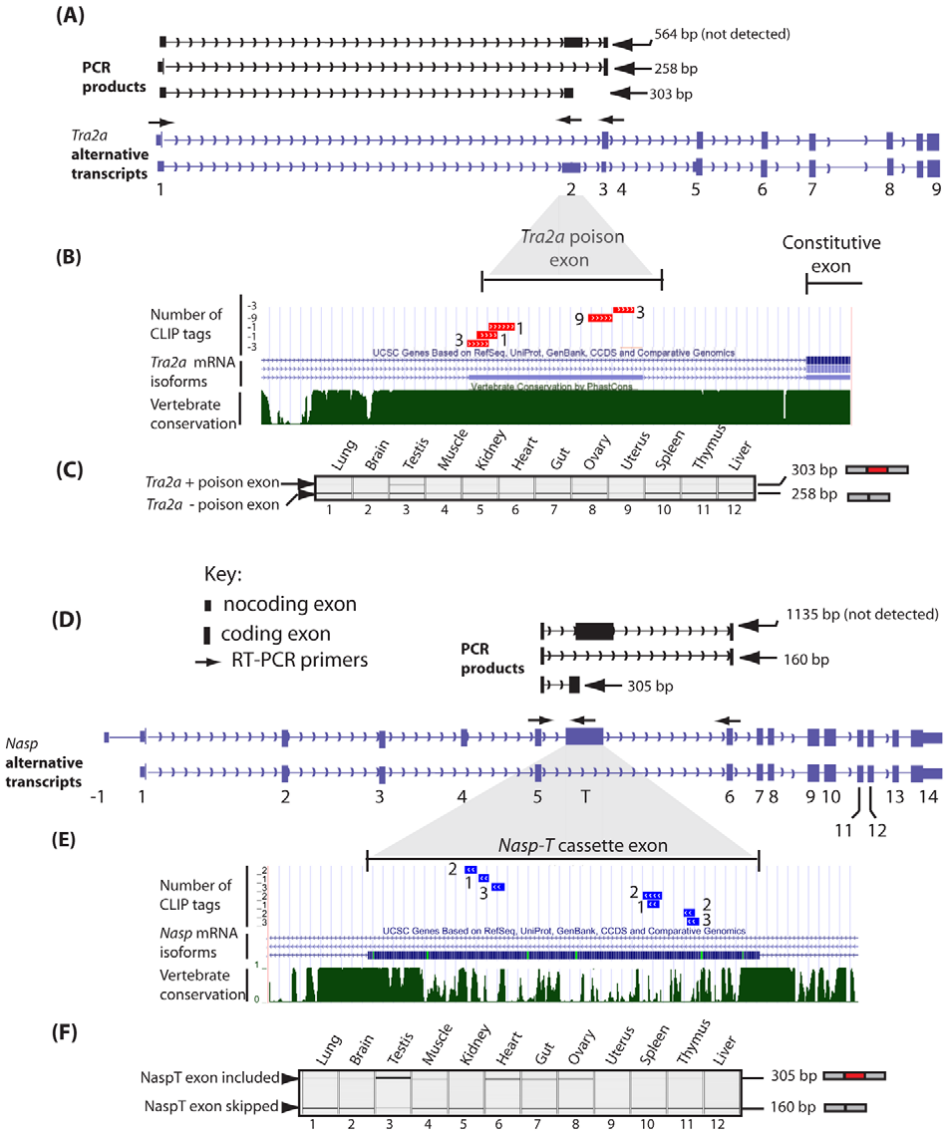
The *Tra2a* poison exon and *Nasp-T* cassette exon are conserved in vertebrates and spliced at high levels of inclusion in the mouse testis. (A) The structure of annotated alternative *Tra2a* transcripts (purple) and predicted PCR products (black) are shown above. (B) Comparative genomic analysis with supporting EST information confirm splicing inclusion of these *Tra2a* poison exons indicate they are found in vertebrates as distantly related as humans, mice, zebrafish and frog. (C) Expression of the *Tra2a* poison exon in different mouse tissues was monitored using RT-PCR (primers in exons 1 and 4) followed by capillary gel electrophoresis, and a representative capillary gel electrophoresis image is shown. (D) Multiple Tra2β CLIP tags mapped to a poison exon in the *Nasp-T* gene. The structure of annotated alternative *Nasp* transcripts (purple) and predicted PCR products (black) are shown above. (E) Underneath the Phastcons alignment of the *Nasp-T* exon from multiple vertebrates is shown. (F) Incorporation of the *Nasp-T* exon was monitored by RT-PCR and capillary gel electrophoresis. High levels of splicing inclusion were detected in the mouse testis, and lower levels of inclusion in other tissues. Multiple CLIP tags mapped to an evolutionarily conserved cassette exon in the *Nasp* gene. The Phastcons alignment of the *Nasp-T* exon from multiple vertebrates is shown. Phastcons analyses in parts (B) and (E) are shown as downloads from UCSC [69]. The key for both parts (A) and (D) are indicated in (D).

An important measure of the functional importance of individual alternative splice events is evolutionary conservation [1], [2], [37], [41], [42]. Although many testis-specific exons are species-specific, phastcons analysis (which measures phylogenetic conservation of sequences on a scale of 0 to 1, with 1 being most conserved) indicated very high levels of phylogenetic conservation for the *Tra2a* poison exon along with flanking intronic sequences (Figure 5A–5C). Similar high levels of nucleotide conservation have been reported for poison exons in other genes encoding splicing regulator proteins including *Sfrs10* itself [4], [5], [22].

The *Tra2a* poison exon, which is 306 nucleotides long, introduces stop codons into the reading frame of the *Tra2a* mRNA which encodes Tra2α protein. Despite the lack of protein coding capacity, 48% of nucleotides within the *Tra2a* poison exon are in fact conserved in all vertebrates (Figure S2A: the nucleotide positions universally conserved in sequenced vertebrate genomes are shown in red). As a group, the 24 top most frequently recovered 6-mers from the entire transcriptome-wide screen were enriched in the nucleotide positions conserved between all vertebrates at levels much higher than would be expected by chance (Figure S2A, p = 0.0075, Fisher exact test: p = 0.0003, Chi Squared test). These data are consistent with maintenance of multiple Tra2β-binding sites within the *Tra2a* poison exon since the radiation of vertebrates. When analysed by RT-PCR, the *Tra2a* poison exon was found to be particularly strongly alternatively spliced in the testis, with zero or much lower levels in other adult tissues (Figure 5A–5C).

Phastcons analyses also showed the *Nasp-T* cassette exon, which is also particularly long at 975 nucleotides, has been conserved since the last common ancestor of all vertebrates (Figure 5D–5F). However neither the nucleotide or the peptide sequence encoded by *Nasp-T* are particularly highly conserved over the full length of the exon (Figure 5E).The *Nasp* gene encodes a histone chaperone essential for mouse development [43], and the *Nasp-T* exon introduces a peptide-encoding cassette exon generating a longer version of the Nasp protein. Similar to the *Tra2a* poison exon, 6-mers predicting Tra2β binding site sequences were found throughout the *Nasp-T* exon, and high frequency 6-mers mapped closely adjacent to CLIP tags (Figure S2B). Within mammalian *Nasp-T* exons multiple Tra2β binding sites have been conserved. Extremely high levels of *Nasp-T* exon inclusion were detected by RT-PCR in the testis and heart. In gut, muscle and ovary, the *Nasp-T* exon inclusion isoform was also preferentially included but in other tissues it was frequently skipped (Figure 5F).

### Efficient splicing activation of the testis-specific Nasp-T by Tra2β depends on multiple Tra2β binding sites

**Figure 6:**
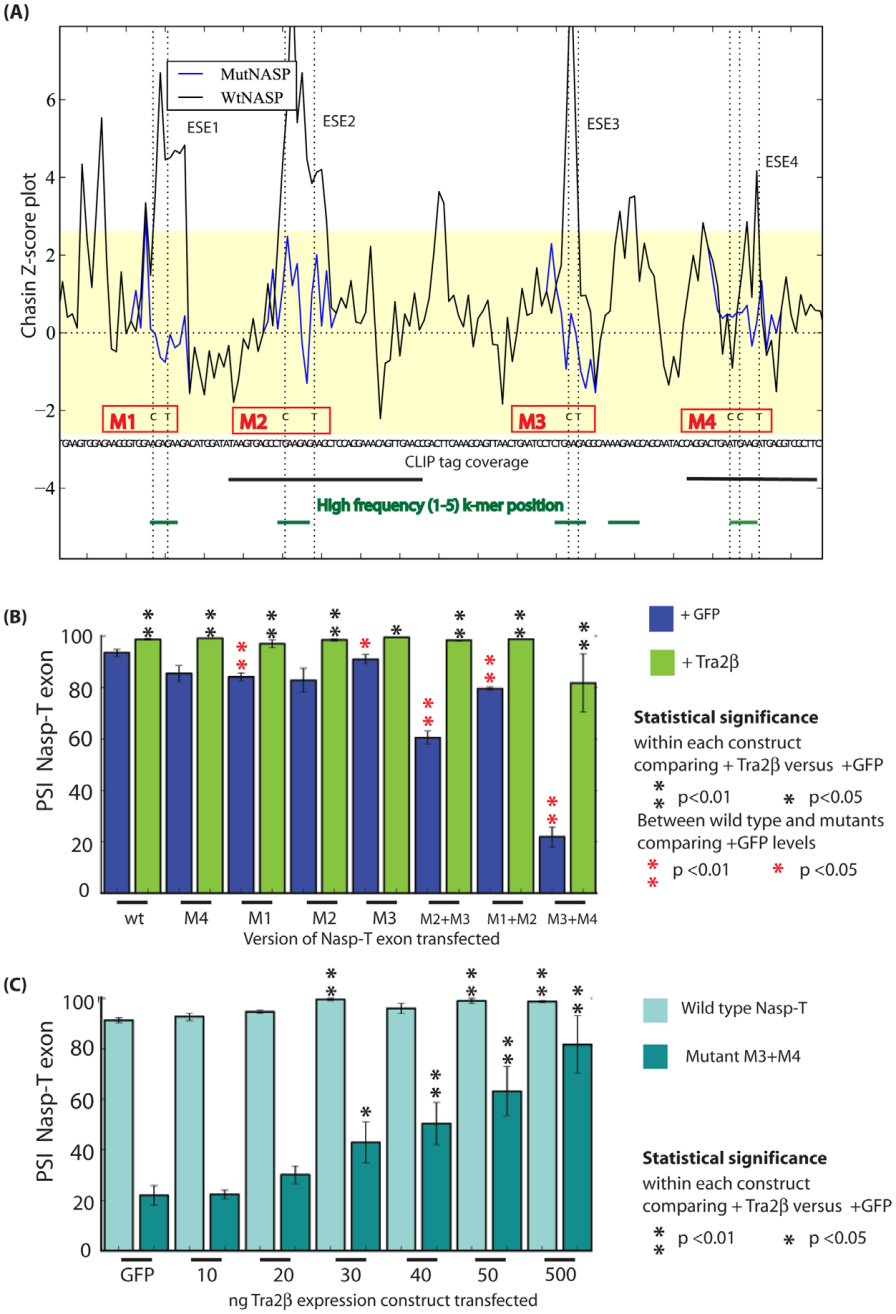
The splicing response to Tra2β is mediated through binding to four independent sites. (A) z-score plot predicting the splicing control sequences according to [45] in the upstream portion of the *Nasp-T* cassette exon. Investigated exonic regions with z-scores above the threshold value for exonic splicing enhancers are labelled ESE1–4. The z-score plots of the wild type *Nasp* exon is shown in black, superimposed with z-score plots for each of the point mutants which affected individual ESEs (shown as blue coloured lines, with the changed nucleotide indicated as a broken line). Individual mutants are shown as M1–M4. Local CLIP tag coverage is shown as black lines, and the relative positions of local 6-mers identified at a high frequency in the CLIP screen as green lines. (B) Effect of Tra2β on splicing inclusion of different *Nasp-T* cassette exons (wild type and mutants) co-expressed in HEK293 cells in the presence of endogenous Tra2β or with constant levels of Tra2β (500 ng, ectopically expressed). (C) Percentage exon inclusion of the wild type and *Nasp-T* exon derivative M3+M4 obtained after transfection of increasing levels of each of Tra2β. Error bars are shown as the standard error of the mean. Probability (p) values were calculated using an independent two-sample T-test between the PSI levels for cells co-transfected with GFP and Tra2β-GFP (black asterisks), or between endogenous PSI for each of the Nasp-T constructs at endogenous Tra2β concentrations (just transfected with GFP, red asterisks). P value scores are indicated as * p≤0.05 and **p≤0.01.

To experimentally address the function of multiple Tra2β binding sites in *Nasp-T* we used a combination of *in silico* and experimental analyses, and focused on an upstream portion of the exon (from positions 117 to 271). Using octamers predictive of splicing enhancers and silencers [44], [45], [46], we firstly identified 3 strong putative ESEs (Exonic Splicing Enhancers, ESE1 to ESE3) which we selected for further analysis, as well as other putative moderate ESEs (Z score around 4) of which only one designated ESE4 was further studied (Figure 6A). Each of these putative ESEs directly overlapped with Tra2β binding sites initially identified through 6-mers derived from the transcriptome-wide CLIP analysis.

To experimentally test the need for individual Tra2β binding sites in splicing regulation, individual sites were mutated within the minigenes without creating Exonic Splicing Silencer (ESS) sequences (Figure 6A) [28], and the splicing effect monitored. Mutation of single Tra2β binding sites had only a minor effect on *Nasp-T* splicing inclusion at endogenous cellular concentrations of Tra2β. However, pre-mRNAs containing double mutations affecting Tra2β binding sites (M2+M3, M1+M2 and M3+M4) had strongly reduced *Nasp-T* exon splicing inclusion compared to their wild type counterparts at normal endogenous cellular concentrations of Tra2β (Figure 6B). Mutation of different Tra2β binding sites within *Nasp-T* also had distinct outcomes on exon inclusion, indicating underlying combinatorial effects between different patterns of Tra2β binding. In particular, mutant M3+M4 reduced exon inclusion levels to 20% of wild type at endogenous cellular levels of Tra2β, whereas double mutations comprising M2 and M3 reduced *Nasp-T* exon inclusion to just below 60% (Figure 6B).

**Figure 7:**
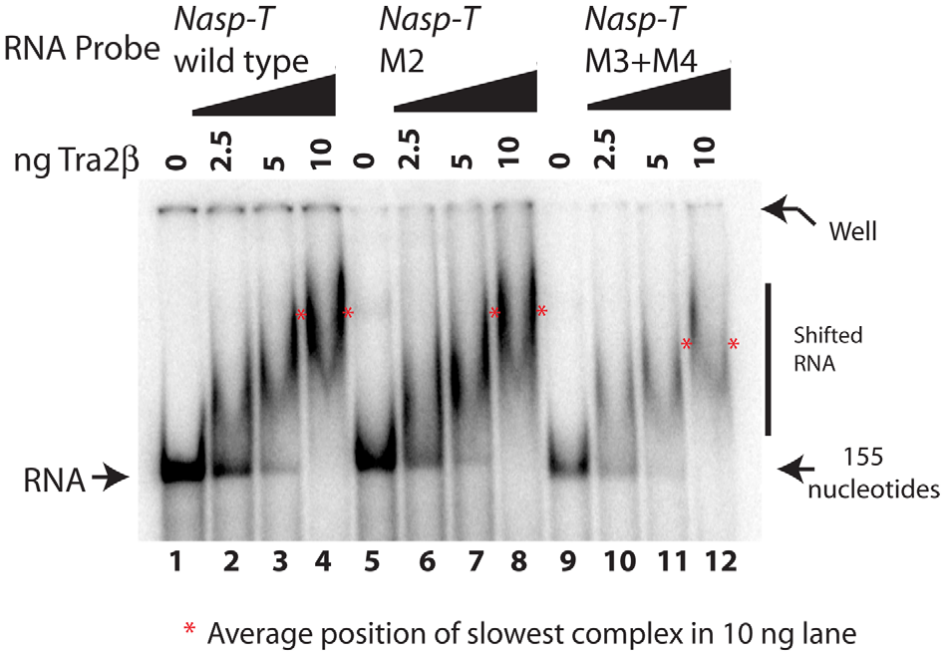
Point mutants in the Nasp-T exon within candidate Tra2β binding sites are still able to bind to Tra2β. RNA-protein interactions were monitored by EMSAs. The average position of the slowest migrating complex in the lane containing 10 ng of added Tra2β protein is indicated by an asterisk, and the RNA probes used were as in Figure 4 but containing the appropriate point mutation.

Although they showed decreased exon inclusion at normal cellular concentrations of Tra2β, each of the double mutated Nasp-T exons gave at least 80% splicing inclusion after Tra2β protein was ectopically expressed. This suggested a requirement for higher levels of ectopic Tra2β protein for splicing inclusion. To test this, we co-transfected cells with minigenes containing either wild type *Nasp-T* exon or the M3+M4 mutant derivative, and a concentration gradient of Tra2β (Figure 6C). Splicing inclusion of the wild type *Nasp-T* exon was already 90% without over-expression of Tra2β and was maximal after co-transfection of no more than 30 ng Tra2β expressing plasmid. In contrast, levels of inclusion of the M3+M4 *NaspT* exon derivative increased more slowly over the whole concentration gradient, indicating decreased splicing sensitivity to Tra2β after removal of just two binding sites. This is particularly striking since the M3+M4 *NaspT* exon retains multiple other Tra2β binding sites (both experimentally confirmed sites in the case of ESEs 1–4, and further predicted sites throughout the exon shown in Figure S1). We used EMSAs to directly analyse RNA-protein interactions using both wild type and mutated versions of the *Nasp-T* RNA probe (Figure 7). While wild type *Nasp-T* and the single mutant M2 RNA were efficiently shifted, the average size of the M3+M4 RNA-protein complex was only slightly smaller (the average size of the shifted complexes is indicated by a red asterisk on Figure 7). Hence even a moderate change in *in vitro* RNA-protein interactions translates to a detectable change in splicing inclusion within cells.

### Levels of neuronal Tra2β protein are depleted in a Nestin-Cre mouse model and are functionally buffered by the *Sfrs10* poison exon

Mice with clearly reduced expression levels of *Sfrs10* would be a prerequisite to enable detection of altered splicing patterns in Tra2β- targeted transcripts identified by CLIP. Since ubiquitous *Sfrs10* deletion leads to embryonic lethality [19], we generated a neuronal specific *Sfrs10*-depleted mouse by crossbreeding *Sfrs10^fl/fl^* mice with *Sfrs10^fl/+^* mice carrying the Nestin-Cre transgene (Nestin-Cre^tg/+^). In *Sfrs10^fl/fl^; Nestin-Cre^tg/+^* offspring the Cre recombinase would be specifically activated in neuronal and glial precursor cells from embryonic day 11 [47] to generate animals with a homozygous *Sfrs10* knockout in the developing central nervous system (CNS).

**Figure 8:**
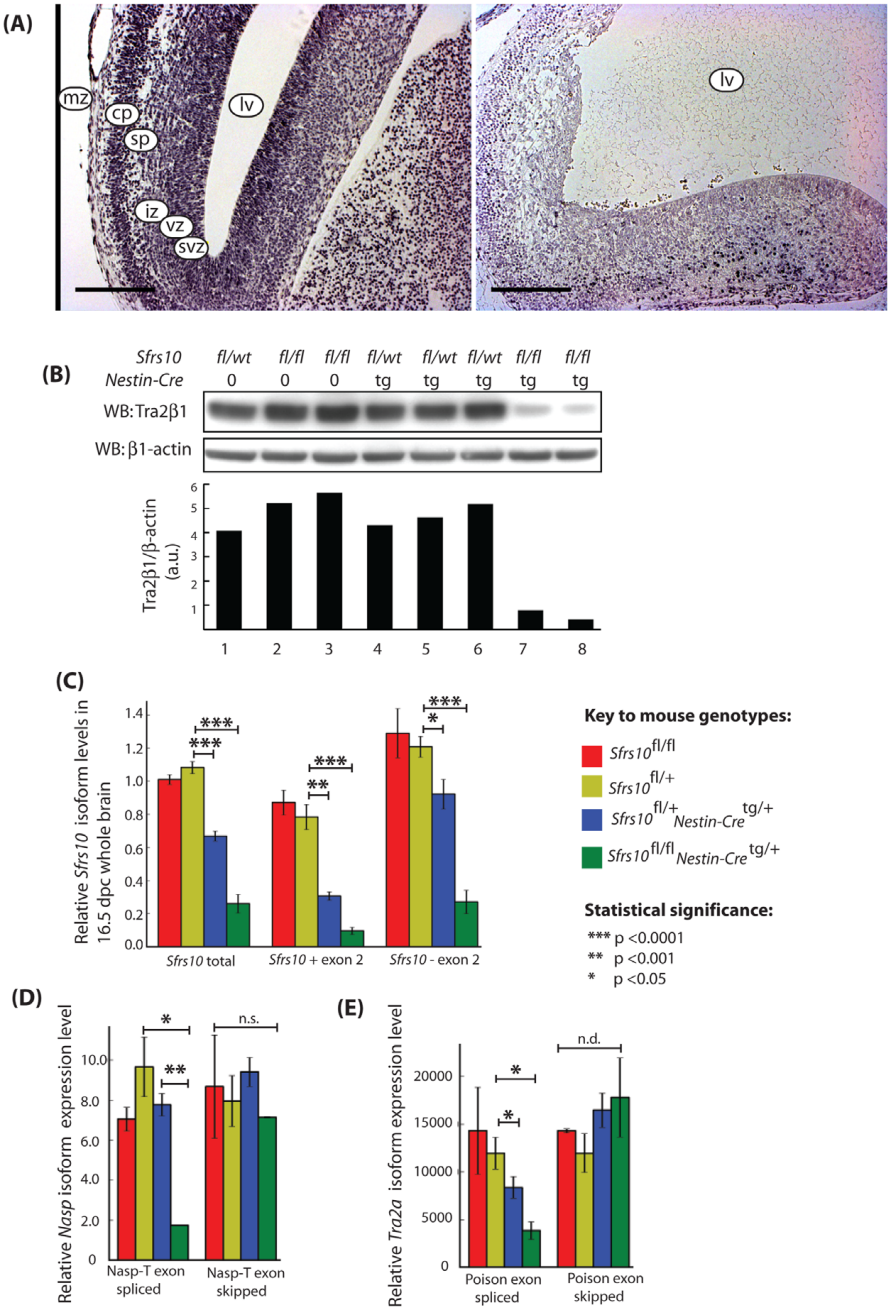
Tra2β protein levels are drastically reduced in the brains of neuronal specific *Sfrs10* knockout mice and correlate with defects in splicing of the *Nasp-T* cassette and *Tra2a* poison exon. (A) Whole brain sections derived from 16.5 dpc *Sfrs10* ^fl/wt^; Nestin-Cre^tg^ (left panel) and *Sfrs10* ^fl/fl^; Nestin-Cre^tg^ (right panel) stained with antibodies against Tra2β. Brains of heterozygous knockout animals (left panel) appear normal and Sfrs10 is expressed throughout all cortical layers. Brains of neuronal specific knockout animals (right panel) show a vast dilation of the lateral ventricles and disturbed cortical patterning. Tra2β expression is not detectable in the majority of intact tissue areas but is clearly retained in some cells of the cortical plate region. Scale bars represent 200 µm. Abbreviations are mz: marginal zone; cp: cortical plate zone; sp: subplate zone; iz: intermediate zone; svz: subventricular zone; vz: ventricular zone; lv: lateral ventricle. (B) Western blot analysis indicates that Tra2β expression is reduced in neuronal specific knockout mice. Proteins were isolated from whole brains of 16.5 dpc embryos and Tra2β was specifically detected by Western blotting. The Tra2β protein level is drastically reduced in *Sfrs10* ^fl/fl^; Nestin-Cre^tg^ animals compared to controls or heterozygous knockout animals. β-actin was used as a loading control. The relative levels are shown underneath as a bar chart (a.u. = arbitrary units). (C) Expression of the *Sfrs10* mRNA in different mouse genotypes used in this study. Levels of the *Sfrs10* mRNA isoforms in different mouse genotypes were independently measured by qRT-PCR from whole brain RNA isolated at 16.5 dpc (*Sfrs10^fl/fl^*, n = 4; *Sfrs10^fl/+^*, n = 5; *Sfrs10^fl/+^; Nestin-Cre^tg/+^*, n = 4; *Sfrs10^fl/fl^*; *Nestin-Cre* ^tg/+^, n = 4). Levels of *Sfrs10* mRNA isoforms are consistent with use of the poison exon for autoregulation of transcript levels in vivo at 16.5 dpc. Isoform-specific qRT -PCR for *Sfrs10* on whole brain RNA revealed a coordinate downregulation of both the functional (−78%) and the non-functional (−88%) isoform in neuronal specific knockout animals at a highly significant level. The decrease of *Sfrs10* transcripts was also detectable in heterozygous knockout animals, in which the functional and non-functional isoform were decreased by 24% and 61%, respectively. (D) Splicing of the *Nasp-T* cassette exon is misregulated in *Sfrs10* ^fl/fl^; *Nestin-Cre^tg/+^*,mice. Levels of the different mRNA isoforms were measured by qRT-PCR from brain RNA samples isolated at 16.5 dpc (*Sfrs10^fl/fl^*, n = 2; *Sfrs10^fl/+^*, n = 3; *Sfrs10^fl/+^; Nestin^−^Cre^tg/+^*, n = 5; *Sfrs10* ^fl/fl^; *Nestin-Cre^tg/+^*, n = 2). (E) Splicing of the Tra2a poison exon is misregulated in *Sfrs10^fl/fl^; Nestin-Cre^tg/+^*mice. Levels of the different mRNA isoforms were measured by qRT-PCR from brain RNA samples (*Sfrs10^fl/fl^*, n = 2; *Sfrs10^fl^* ^/+^, n = 3; *Sfrs10^fl/+^; Nestin-Cre^tg/+^*, n = 5; *Sfrs10^fl/fl^; Nestin-Cre^tg/+^*, n = 2). (C–E) Error bars represent the s.e.m. Statistical significance was monitored using the T-test, and the significance values are as indicated.

Homozygous neuronal *Sfrs10* mice died immediately after birth at postnatal day 1 (PND1) whereas heterozygote mice had normal lifespans. Neuronal specific *Sfrs10*-depleted embryos showed severe malformations of the brain including strong dilation of the third and lateral ventricles as well as degeneration of cortical structures (Figure 8A, right panel and data not shown) whereas heterozygous knockout mouse embryos (Sfrs10^fl/wt^; Nestin-Cre^tg^) had normal brain morphology (Figure 8A, left panel). This indicates Tra2β protein is functionally very important for brain development in the mouse. As the liquid filled ventricles make up the majority of the whole brain volume, the brain morphology is heavily altered and the proportion of intact tissue is heavily reduced. Immunohistochemical analysis of whole brain paraffin-embedded cross-sections showed strongly decreased expression of Tra2-β with some Tra2-β positive cell areas in the cortical plate zone (Figure 8A, right panel). These residual Tra2-β positive cells likely do not express *Cre* from the *Nestin* promoter and are likely of non-neuronal origin, or may represent mosaicism of *Nestin-Cre* expression. Furthermore, Western blots from whole brain also demonstrated a clear down-regulation of Tra2-β in neuronal specific *Sfrs10*-depleted embryos compared to controls and heterozygous knockout animals at 16.5 dpc (Figure 8B). In control animals the *Sfrs10* mRNA levels remained largely unchanged during development (16.5 dpc, 18.5 dpc and PND1) (*Sfrs10* ^fl/fl^ n = 10; *Sfrs10* ^fl/+^ n = 6; data not shown).

Expression analysis of whole brain RNA from neuronal *Sfrs10*-depleted embryos at 16.5 dpc and 18.5 dpc and mice at PND1 showed clearly reduced *Sfrs10* mRNA levels compared with brains of control littermates (*Sfrs10^fl/fl^*, *Sfrs10^fl/+^* or *Sfrs10^fl/+^; Nestin-Cre^tg/+^*) (Figure 8C). Regardless of the developmental stage the majority of *Sfrs10^fl/fl^* pups exhibited somewhat reduced *Sfrs10* expression levels compared with heterozygously floxed mice, which suggested that the integration of the floxed allele has a slightly negative influence on *Sfrs10* expression. Therefore for statistical analysis the expression levels of splice isoforms of *Sfrs10^fl/fl^*; *Nestin-Cre^tg/+^* mice were always compared with *Sfrs10* ^fl/+^ and not *Sfrs10^fl/fl^* mice.

Tra2-β regulates its own expression level via alternative splice regulation in an autoregulatory feedback-loop. Inclusion of poison exon 2 into *Sfrs10* transcripts introduces a premature stop codon which leads to a non-functional protein and thus a reduction in Tra2-β levels [22]. Isoform specific qRT-PCR indicated a highly significant down-regulation of both individual mRNA splice isoforms and total length *Sfrs10* mRNA in neuronal specific *Sfrs10*-depleted mice Sfrs10^fl/fl^Nestin-Cre^tg/+^) compared to controls at 16.5 dpc (Figure 8C). In contrast, in heterozygous knockout animals (Sfrs10^fl/+^Nestin-Cre^tg/+^) down-regulation of the functional isoform (− exon 2) was less effective than for the non-functional (+ exon 2) isoform indicating the involvement of the autoregulatory feedback loop which counteracts any decrease in functional Tra2β protein in neuronal cells.

### Tra2β physiologically regulates splicing inclusion of the *Tra2a* poison and *Nasp-T* cassette exons in mouse brain development

We next set out to determine whether the *Tra2a* poison exon and *Nasp-T* cassette exon were true physiological target exons regulated by Tra2β *in vivo*. Correlating with an important regulatory role for Tra2β protein, splicing inclusion of the poison exon into the *Tra2a* mRNA was reduced 3-fold in neuronal *Sfrs10*-depleted mouse brains compared to controls at 16.5 dpc (Figure 8E). Surprisingly, this decrease in poison exon inclusion could not be detected at later developmental stages like 18.5 dpc or PND1 (data not shown).

To determine whether low Tra2β levels directly affect the splicing of the *Nasp-T* exon, qRT-PCR was carried out on whole brain RNA of 16.5 dpc and PND1 pups. The levels of the T-exon isoform of *Nasp* mRNA (*Nasp-T*) were 4-fold reduced in brains of neuronal *Sfrs10*-depleted mice compared to controls at 16.5 dpc (Figure 8D) and PND1 (data not shown). Given the 4-fold reduction of the *Nasp-T* isoform in *Sfrs10*-depleted tissue, we conclude that Tra2β protein is likely to be an important *in vivo* activator of *Nasp-T* exon inclusion during mouse development.

These data correlate a defect in splicing regulation of *Nasp-T* and *Tra2a* with *Sfrs10* depletion but do not necessarily imply a causal relationship, because of the differences in cell types present after *Sfrs10* depletion which result from the physiological importance of Tra2β for brain development. To address this further we compared overall patterns of expression of the *Nasp* and *Tra2a* genes in wild type and knockout mice, by quantifying levels of the somatic *Nasp* and *Tra2a* mRNA isoforms. Consistent with no significant changes in overall *Tra2a* gene expression resulting from changes in the cell type population of the knockout brains, no statistically significant changes in functional *Tra2a* or *Nasp* expression were seen when comparing brain RNA of *Sfrs10^fl/+^* mice with RNA of *Sfrs10^fl/fl^; Nestin-Cre^tg/+^* mice (Figure 8D and 8E). These results are consistent with essentially similar patterns of *Nasp* and *Tra2a* gene expression in the mutant and wild type brains despite any differences in cellular composition, while in contrast the Tra2β-regulated splice isoforms from these same genes are very different between the wild type and mutant mice.

## Discussion

Here we have identified (for the first time to the best of our knowledge) physiological target exons regulated by Tra2β during mouse development. Identification is based on the criteria of *in vivo* cross-linking of endogenous RNAs and proteins, *in cellulo* experiments using transfected minigenes and proteins, RNA-protein interaction assays and genetic analysis using a newly derived conditional mouse strain which does not express Tra2β protein in neurons and has significant abnormalities in brain development. Our analyses reveal important pathways regulated by Tra2β protein *in vivo* which likely contribute both to prenatal death in *Sfrs10* ^−/−^ embryos and also to normal germ cell development [19]. Nasp protein is a histone chaperone required for nuclear import of histones at the G1-S phase transition of the cell cycle, and is essential for cell proliferation and embryonic survival [43]. Nasp functions in chromatin remodelling after DNA repair, and links chromatin remodelling to the cell cycle machinery after S phase [48]. The T exon is also spliced in embryos, and within the testis alternative splicing inclusion of the *Nasp-T* cassette exon generates the testis-enriched tNASP protein isoform. Timing of tNASP protein expression during male adult germ cell development [48], [49] exactly parallels the expression of Tra2β protein. The tNASP protein isoform localises to the synaptonemal complex of meiotic chromosomes where it may help monitor double strand DNA break repair [43], [48], [50].

Tra2α and Tra2β are very similar proteins, and are interchangeable in our *in cellulo* splicing assays. Tra2β protein helps regulate overall Tra2 protein levels through both activating splicing inclusion of a poison exon into its own *Sfrs10* mRNA, and also activating splicing inclusion of a poison exon into *Tra2a* mRNA which encodes Tra2α protein. *In vivo* experiments described here show that reduced inclusion of the poison exon does indeed help buffer the effect of decreased gene dosage in *Sfrs10* heterozygote mice. However, down-regulation of *Tra2a* poison exon inclusion in *Sfrs10^−/−^* cells does not lead to an increase in *Tra2a* mRNA levels sufficient to restore splicing patterns of Tra2β target exons, perhaps suggestive of unique functions for the Tra2α and Tra2β proteins. In flies, auto-regulation of splicing by Tra2 protein of its own pre-mRNA has been shown to be critical for spermatogenesis, indicating that it might be a highly conserved feature for germ cells to tightly maintain expression levels of this class of splicing regulator [24], [25], [51]. Since Tra2α regulates *Tra2a* poison exon *in cellulo*, it is likely that it also autoregulates its own mRNA levels *in vivo* through activation of this same poison exon.

An important current question is how RNA binding proteins like Tra2β achieve sequence specificity in target sequence selection despite having fairly short target sequences [15]. Here we have found a short consensus binding motif for Tra2β (AGAAGA, Figure 2A) which matches perfectly with specific motifs obtained both by classical SELEX analysis [12] and from identification of Tra2β specific ESEs in various genes [22], [29], [52], [53], [54], [55], [56], [57]. Parallel genome-wide mapping showed that Tra2β primarily binds to exonic sequences. An explanation for exonic enrichment despite the short binding site would be if Tra2β binds to exons cooperatively with adjacent exonic RNA binding proteins. In the case of *SMN2* exon 7, the Tra2β binding site is flanked by cooperative binding sites for SRp30c and hnRNP G [17], [53], [58]. For *Nasp-T* and *Tra2a* there are instead arrays of exonic Tra2β binding sites. Removal of more than one binding site negatively affects exon activation by Tra2β, indicating for *Nasp-T* and *Tra2a* adjacent binding and assembly of homotypic Tra2β protein activation complexes play important roles in splicing activation.

A model of splicing activation for the *Nasp-T* and the *Tra2a* poison exon which depends largely on sole binding of Tra2β protein might explain why these exons are particularly sensitive to depletion of Tra2β *in vivo* compared with *SMN2* exon 7 (splicing of which is not affected after deletion of *Sfrs10*, and which has a single Tra2β binding site, Figure S1). The human testis-specific HIPK3-T exon [50] also requires multiple Tra2β binding sites to enable splicing activation of a weak 5′ splice site *in vitro* [28], and the *Sfrs10* poison exon also has multiple Tra2β binding sites [22]. Other than *Tra2a* and *Nasp-T*, the remaining target exons we analysed using minigenes here have less dense coverage of Tra2β binding sites (Figure S1). These remaining exons also responded less robustly to Tra2β protein expression *in vitro* in transfected cells, and it is likely that RNA binding proteins other than Tra2β might also be more important for their splicing regulation *in vivo*.

We also found that full lengthTra2β protein activates splicing of the *Nasp-T* exon at a lower level through its RS1 and RS2 domains only (i.e. without the RRM and so without direct RNA binding). Mechanistically the RS domains of Tra2β might activate splicing by helping assemble other RS-domain containing splicing regulators and components of the spliceosome into functional splicing complexes. Although both RS domains could co-activate splicing when present together, removal of the RS1 domain completely disabled Tra2β-mediated splicing activation of the physiological target exons identified here. The observed functional importance of RS1 provides a mechanistic explanation why this N-terminal RS domain structure is maintained for Tra2 proteins in both vertebrates and invertebrates. Surprisingly Tra2β molecules without the RS1 domain were not just neutral for splicing inclusion *in cellulo*, but for some exons actually functioned as potent splicing repressors. Since the Tra2βΔRS1 isoform contains a functional RRM sequence, splicing repression could be due to competitive inhibition through this shorter Tra2β protein binding to the same RNA targets, but then being unable to assemble functional splicing complexes with other Tra2β proteins in the absence of the RS1 domain. Detection of such a competitive inhibitory function might have been helped by the increased levels of the Tra2βΔRS1 isoform expressed in our experiments. *In vivo*, the Tra2β-3 protein which lacks the N-terminal RS1 domain might also operate as a natural splicing repressor isoform [20], [21], [22], depending on its level of expression being enough in specific cell types or tissues. Tra2βΔRS1 actually activates *SMN2* exon 7 rather than being a repressor as seen for the physiological target exons we describe in this report [17]. Although the biology of *SMN2* exon 7 has been an area of controversy in the literature [59], [60], a possible mechanistic explanation for this difference might be if Tra2β binding to *SMN2* exon 7 blocked the action of an adjacent Exonic Splicing Silencer, rather than directly activating splicing by itself.

Our analysis shows that the RNA targets identified for Tra2β in developing adult germ cells can predict patterns of splicing regulation by Tra2β in the developing brain. However, our data further suggest that splicing regulation by Tra2β is temporally restricted during development and also differentially regulated between various Tra2β targets. This is highlighted by *Tra2a* poison-exon splicing, which is affected by neuronal specific *Sfrs10* knockout only at a defined developmental stage, while *Nasp-T* exon inclusion is perturbed by *Sfrs10* knockout in all analyzed situations. Both the *Nasp-T* and the *Tra2a* poison exon are biologically important: they are conserved in all vertebrates for which genome sequences are available; have known functional roles; and like other phylogenetically conserved exons are spliced at high levels in at least some tissues [4], [37], [41]. The tNASP protein has been identified immunologically after the leptotene stage of meiosis in both rabbits and mice, indicating that this exon is meiotically expressed in both species [48], [49]. In addition, although a high frequency of alternative splicing events in the testis are species-specific [61], the high conservation of binding sites in the *Tra2a* poison-exon suggests regulation by Tra2β has been conserved since the radiation of vertebrates. Overall our data indicate maintenance of ancient patterns of splicing regulation controlled by this RNA binding protein, consistent with its observed key role in development [19].

## Materials and Methods

### Detection of RNA and proteins in different mouse tissues

mRNA levels were detected in total RNA isolated from different mouse tissues using RT-PCR and standard conditions. RT-PCR products were analysed both by normal agarose gel electrophoresis (not shown) and capillary gel electrophoresis [62], [63]. *Sfrs10* primers were specific to sequences in exons 1 and 4 respectively (5′-GAGCTCCTCGCAAAAGTGTG-3′ and 5′-CAACATGACGCCTTCGAGTA-3′). Tra2β protein was detected using immunohistochemistry in the mouse brain as previously described [64] and in the mouse testis using Abcam polyclonal Tra2β antibody ab31353 [28] as previously described [26].

Different *Tra2a* mRNA isoforms mRNA were detected by multiplex RT-PCR using Tra2aF (5′-GTTGTAGCCGTCGCCTTC T-3′), Tra2aB (5′-TGGGATTCAGAATGTTTGGA-3′) and Tra2a poison (5′-TTCAAGTGCTTCTATCTGACCAA-3′). Different *Nasp-T* mRNA isoforms were detected by RT-PCR using Nasp-TF (5′-AATGGAGTGTTGGGAAATGC-3′), Nasp-TB (5′-TTGGTGTTTCTTCAGCCTTG-3′) and Nasp-TC (5′-TGCTTTGAAGTCGGTTCAACT-3′).

*Hprt* expression was detected using primers HrptF (5′-CCTGCTGGATTACATTAAAGCACTG-3′) and HprtR (5′-GTCAAGGGCATATCCAACAACAAAC-3′).

### HITS-CLIP

HITS-CLIP was performed as previously described [30] using an antibody specific to Tra2β [65]. The specificity of the antibody to Tra2β was confirmed by the experiment shown in Figure S3, as well as the additional characterization already described [65]. In short, for the CLIP analysis mouse testis was sheared in PBS and UV crosslinked. After lysis, the whole lysate was treated with DNase and RNase, followed by radiolabelling and linker ligation. After immunoprecipitation with purified antisera specific to Tra2β [65], RNA bound Tra2β was separated on SDS-PAGE. A thin band at the size of 55 kDa (Tra2β migrates at around 40 kDa and MW of 50 nt RNA is about 15 kDa) was cut out and subject to protein digestion. RNA was recovered and subject to sequencing which was carried out on the Newcastle University Roche 454 GS-FLX platform. Mapping was done with Bowtie [66], allowing for two mismatches (parameter –v 2). 297070 reads were processed, of which 177457 (59.74%) aligned successfully to the mouse genome (Mm9). 74476 (25.07%) failed to align, and 45137 (15.19%) reads were suppressed due to multiple hits on the mouse genome. K-mer analysis was carried out using custom written scripts in Python. Briefly, we calculated the frequency of occurance of each possible 6-mer sequence in the following: our CLIP dataset, the mouse genome (mm9) and in the mouse testis transcriptome (http://www.ncbi.nlm.nih.gov/projects/geo/query/acc.cgi?acc=GSM475281). The genome and transcriptome corrected frequencies were obtained by subtracting the background (genome and transcriptome frequencies respectively) from the signal (frequency in CLIP dataset). CLIP reads were filtered to remove duplicates including overlapping reads. Statistical significance was determined using a Chi-squared test. The weblogo was derived from tags containing a GAA sequence by analysing the sequence composition surrounding the fixed sequence, using custom written scripts to generate an input for the freely available program weblogo (http://weblogo.berkeley.edu/).

### Generation of neuronal specific *Sfrs10* knock-out mice for in *vivo* splicing analysis

In our *in vivo* splicing study we utilized a previously established *Sfrs10* mouse model on pure C57BL/6 background as described [19]. Genotyping was performed using tail DNA according to established protocols [19]. To induce a conditional *Sfrs10* knock-out in the central nervous system we crossbred *Sfrs10^fl/fl^* mice with a *Nestin-Cre^tg/+^* mouse line. These mice express Cre recombinase under control of the rat nestin (Nes) promoter and enhancer [47]. Therefore Cre recombinase is expressed in neuronal and glia cell precursors from embryonic day 11 as well as in neurogenic areas of the adult brain [47], [67]. For our analyses the presence of the *Nestin* transgene was determined by a standard PCR using the oligonucleotides 5′–CGCTTCCGCTGGGTCACTGTCG-3′ (forward) and 5′–TCGTTGCATCGACCGGTAATGCAGGC-3′ (reverse) at an annealing temperature of 58°C producing a 300 bp amplicon.

### Quantitative analysis of *Sfrs10* expression and Tra2β targeted transcripts

Whole brain RNA was isolated from 16.5 dpc, 18.5 dpc and PND1 mice using the RNeasy Lipid Tissue Mini Kit (Qiagen, Hilden, Germany). RNA concentration was determined by Quant-iT RiboGreen RNA Reagent and Kit (Invitrogen, Darmstadt, Germany) and equal amounts of RNA were used for first strand cDNA synthesis utilizing the QuantiTect reverse Transcription Kit (Qiagen, Hilden, Germany). Quantitative real-time PCR was carried out using the Roche LC FastStart DNA Master SYBR green Kit (Roche, Mannheim, Germany) on the Roche LightCycler 1.5. For realtime quantification total *Sfrs10* transcripts were amplified using the oligonucleotides 5′-TAGAAGGCATTATACAAG-3′ (forward) and 5′′-CTCAACCCAAACACGC-3′ (reverse) at 3 mM MgCl_2_ and an annealing temperature of 63°C producing a 186 bp bp amplicon. To quantify *Sfrs10* isoforms specifically we used the oligonucleotides 5′-AGAACTACGGCGAGCGGGAATC-3′ (forward) and 5′-CCTTGTATAATGCCTTCTAGAACTTCTTC-3′ (reverse) for the functional isoform and 5′-GAACTACGGCGAGCGGGTTAATG-3′ (forward) and 5′-CAAGTGGGACTTCTGGTCTGATAATTAGC-3′ (reverse) for the non-functional isoform. Both were run at annealing temperatures of 64°C resulting in amplicons of 191 bp and 161 bp, respectively. For the quantification of different target splice variants single isoforms were amplified separately. For the *Nasp-T* exon containing isoform the oligonucleotides 5′-GGAGTGCATGTAGAAGAGG-3′ (forward) and 5′-CGTCATAAACCTGTTCTCTC-3′ (reverse) were used at 1 mM MgCl_2_ and annealing at 65°C producing a 115 bp amplicon. The somatic isoform of *Nasp* was amplified using 5′-AATGGAGTGTTGGGAAATGC-3′ (forward) and 5′-CTGAGCCTTCAGTTTCATCTAC-3′ (reverse) at 3 mM MgCl_2_, 62°C annealing while producing a product of 118 bp length. The functional *Tra2a* transcript was amplified using the oligonucleotides 5′-GTTGTAGCCGTCGCCTTCT-3′ (forward) and 5′-GAGACTCTCTGCCCTCGAAG-3′ (reverse) at 3 mM MgCl_2_ and 66°C annealing resulting in a 155 bp product. For the poison exon-containing isoform we used the same forward oligonucleotide as for the functional isoform and 5′-CTTGATTTATCTTCCACATTCTTGG-3′ (reverse) at 3 mM MgCl_2_ and 64°C annealing producing a 206 bp amplicon. All quantification data was normalized against *Gapdh*. Amplification was performed using the oligonucleotides 5′-GGCTGCCCAGAACATCATCC-3′ (forward) and 5′-GTCATCATACTTGGCAGGTTTCTC-3′ (reverse) at 3 mM MgCl_2_ and 63°C annealing producing a 169 bp amplicon. Agarose gel electrophoresis and basic melting curve analysis was performed to confirm PCR product specificity. For quantification a dilution series of cDNA was used to generate a standard curve for each isoform. Therefore the cycle threshold was plotted versus the logarithm of the concentration and the standard curve was determined by linear regression. This curve was then utilized to calculate the template concentration of unknown samples. All samples were measured in duplicates. Individuals of a genotype were averaged using the arithmetic mean. Fluctuations are displayed by the standard error of the mean, and these are indicated on the bar charts by error bars. The significance of differences between genotypes was verified using student's t-test.

### Minigene splicing experiments

Candidate alternatively spliced exons identified by HITS-CLIP and approximately 240 nucleotides of intronic flanking region at each end were amplified from mouse genomic DNA with the primer sequences given below. PCR products were digested with the appropriate restriction enzyme and cloned into the *Mfe*1 site in pXJ41 [68], which is exactly midway through the 757 nucleotide rabbit β-globin intron 2. PCR products were made using the following primers:

Krba1L: 5′-AAAAAAAAGAATTCtggggatcctagcaggtaca -3′

Krba1R: 5′-AAAAAAAAGAATTCccaaggatgtgataagcagga -3′

CREB2U: 5′-AAAAAAAACAATTGgggaccattcctcatttcct -3′

CREB2D: 5′-AAAAAAAACAATTGaaggcagttgtcatcattgc -3′

LIN28F: 5′-AAAAAAAAGAATTCccagcctggtctttaagagagt -3′

LIN28B: 5′-AAAAAAAAGAATTCcatacagtgaattatttgaaaacacc -3′

PankF: 5′-AAAAAAAAGAATTCcacatctgtgggtgcacttt -3′

PANKR: 5′-AAAAAAAAGAATTCttcaaaggactatttggttaacagc -3′

FABP9F 5′-AAAAAAAACAATTGtggcattcctttctcacctt -3′

FABP9R 5′-AAAAAAAACAATTGgagccttcctgtgtgggtat -3′

CREBGammaF: 5′-AAAAAAAACAATTGcaaacttctagatggtagaatgatagc -3′

CREBGammaR: 5′-AAAAAAAACAATTGtagccagagaacggaaccac -3′

NaspTF: 5′-AAAAAAAACAATTGtccttggaggacttctgttttc-3′

NaspTR: 5′-AAAAAAAACAATTGggcatgcctgcttaagtgta-3′

Tra2aF: 5′-AAAAAAAAGAATTCattagggactaggatggaacatga -3′

Tra2aR: 5′-AAAAAAAAGAATTCgcatgatggcacatgacttt-3′

ESE mutations within Nasp-T were made by overlap PCR with the additional primers NASPM1-S (5′-GGGTGGACGATAAGACAT GG-3′) and its complementary primer (5′-CCATGTCTTATCGTCCAC CC-3′); NASPM2-S (5′-GTGAGCCTCAAGAGTAGCTCC-3′) and its complementary primer 5′-GGAGCTACTCTTGAGGCTCAC-3′; NASPM3-S (5′-GAATCCTCTGCATAGGCAAAAG-3′) and its complementary primer (5′-CTTTTGCCTATGCAGAGGATT C-3′); NASPM4-S (5′-GGACTGACTCAAGTTGAGGTCGC-3′) and its complementary primer (5′-GCGACCTCAACTTGAGTCAGTCC-3′).

Analysis of splicing of pre-mRNAs transcribed from minigenes was carried out in HEK293 cells as previously described using primers within the β-globin exons of pXJ41 [29]. Because of the length of the regulated exons, additional internal primers were included in multiplex to detect inclusion of the Nasp-T cassette exon (5′-TGCTTTGAAGTCGGTTCAACT-3′) and Tra2a poison exon (5′-TTCAAGTGCTTCTATCTGACCAA-3′).

### EMSAs

EMSAs were carried out as previously described [28] using full length Tra2β protein and in vitro translated RNA probes made from constructs containing amplified regions of the mouse genome cloned into pBluescript. Regions of the mouse genome were amplified using the following primers:

Nasp1TraGSF 5′-AAAAAAAAGGTACCGAAGTGGAGAAGGGTGGAAG-3′

Nasp1TraGSB 5′-AAAAAAAAGAATTCGAAGCGACCTCATCTTCATTC-3′

Krba1GSF 5′-AAAAAAAAGGTACCGACTCCTCCCCACCCTAGTC-3′

Krba1GSR 5′-AAAAAAAAGAATTCGCCCAGCCATCTTCTACCTT-3′

Tra2aGSF 5′-AAAAAAAAGGTACCTTAATGTTCGTGAAGAAATTGAAGAG-3′

Tra2aGSR 5′-AAAAAAAAGAATTCTCATTAGCCTTCTTTTATCTTGATTTA-3′

Lin28GSF 5′-AAAAAAAAGGTACCCTTGAACTCTCTGATTTTAGGTTCTTC-3′

Lin28GSR 5′-AAAAAAAAGAATTCAACAGACTAACCTGGGGCTGA-3′

CrebγF 5′-AAAAAAGGTACCTCATTGTTCTAGGTGCTATCAAAGG-3′

CrebγR 5′-AAAAAAGAATTCCTGACATATTTTATTTTCTCATAGTAT GTCTCTC-3′

Creb2F 5′-AAAAAAGGTACCGTAACTAAATGACCATGGAATCTGGAGCA-3′

Creb2R 5′-AAAAAAGAATTCCTGGGCTAATGTGGCAATCTGTGG-3′

## Supporting Information

Dataset S1 BED file containing the Tra2β CLIP tag sequences and their location in the mouse genome (mm9). This bed file can be saved and added as an optional track on the UCSC mouse genome browser (http://genome.ucsc.edu/). To load this BED file on the UCSC genome browser, use the “manage custom tracks” button under genomes. Alternatively, the bed file can be visualised by up loading the link http://research.ncl.ac.uk/ElliottGroup/UCSC/hub.txt into the My Hubs textbox in the UCSC Track Hubs menu.
(TXT)

Figure S1 Sequence of all the exons analysed using minigenes and some known Tra2β target exons. The Tra2β binding sites predicted from the k-mer analysis are coloured as indicated in Table S1.
(DOC)

Figure S2 Multiple Tra2β binding sites are phylogenetically conserved in *Tra2a* poison exons and Nasp-T exons. (A) Sequence of the *Tra2a* poison exon from mouse. (B) Sequence of Nasp-T exon from mouse. Nucleotides in red are conserved in all vertebrates analysed (mouse, frog, rabbit, human, rat, cow, orang-utan, chimp, macaque, marmoset, guinea pig, dog, horse, elephant, opossum, lizard, zebrafinch, tetraodon, stickleback, medaka, chicken). Nucleotides conserved in all mammals are shown in blue. All other nucleotides are shown in black. The Tra2β binding sites predicted from the k-mer analysis are shaded as indicated in Table S1, and the positions of CLIP tags are underlined (note that some of these underlined regions correspond to multiple overlapping CLIP tags which have been joined in this figure).
(DOC)

Figure S3 Experiment to confirm the specificity of the polyclonal antisera used for CLIP analysis. HEK293 cells were transfected with plasmids expressing the indicated proteins, proteins isolated and analysed by SDS-PAGE and Western blotting. The same blot was probed sequentially with an affinity purified antisera raised against Tra2β [65] and then with a polyclonal specific for GFP to detect expression of the fusion proteins. The affinity purified α-Tra2β antisera detected a single band in HEK293 cells corresponding to endogenous Tra2β protein, and also the Tra2β-GFP fusion protein. No recognition of either Tra2α or Tra2βΔRS1-GFP was observed, indicating that this antisera is highly specific.
(TIF)

Table S1 Properties of the 30 most frequently retrieved 6-mers in the Tra2β CLIP tags. The 6-mers are ordered from the most frequently recovered at the top of the table (AGAAGA) to the 34^th^ most frequently recovered 6-mer at the bottom (GAAGCT). The 6-mers are arranged in colour blocks of 5 according to their frequency of retrieval, and compared and corrected with their frequencies in both the total mouse genome and mouse testis transcriptome. The same colour code of the different 6-mer categories are also used to illustrate the occurrence of these 6-mers within the Tra2β target exons in Figures S1 and S2.
(DOC)

Table S2 List and properties of all 6-mers recovered by Tra2β CLIP above background levels.
(XLSX)

Table S3 Top functions associated with Tra2β-bound mRNAs determined from Ingenuity Pathway Analysis (IPA).
(DOCX)
