# Supplementary material for: Identification of Evolutionarily Conserved Exons as Regulated Targets for the Splicing Activator Tra2β in Development
Source: PLoS Genet. 2011 Dec 15;7(12):e1002390. doi: 10.1371/journal.pgen.1002390 (PMC3240583; doi:10.1371/journal.pgen.1002390)
Supplement: Figure S1 — Sequence of all the exons analysed using minigenes and some known Tra2β target exons. The Tra2β binding sites predicted from the k-mer analysis are coloured as indicated in Table S1. (DOC) [file pgen.1002390.s002.doc]

**Figure S1**

**Exons tested by mini-gene constructs for response to Tra2β**

Putative Tra2β binding sites represented by k-mers are highlighted as follows:

NNN: K-mer Ranking 1-5

NNN: K-mer Ranking 5-10

NNN: K-mer Ranking 10-15

NNN: K-mer Ranking 15-20

NNN: K-mer Ranking 20-25

**NASP-T exon**

AGGAAGCAAGGGAAGAGTTGAGAGAACAGGTTTATGACGCCATGGGAGAAAAAGAAGCCAAAAAAGCAGAAGGCAAGTCTCTGACAAAGCCTGAAACTGATAAAGAACAGGAAAGTGAAGTGGAGAAGGGTGGAAGAGAAGACATGGATATAAGTGAGCCTGAAGAGAAGCTCCAGGAAACAGTTGAACCGACTTCAAAGCAGTTAACTGAATCCTCTGAAGAGGCAAAAGAAGCAGCAATACCAGGACTGAATGAAGATGAGGTCGCTTCTGGGAAGACAGAGCAGGAATCATTGTGTACTGAGAAAGGAAAATCAATTTCAGGAGCTTATGTTCAAAATAAAGAATTCAGAGAAACAGTGGAGGAGGGAGAGGAAATAATAAGCTTAGAGAAAAAGCCAAAAGAAACTTCAGAAGATCAGCCTATCAGGGCTGCAGAAAAGCAGGGCACTTTAATGAAGGTGGTAGAAATAGAAGCTGAAATAGACCCTCAAGTCAAGTCAGCGGATGTGGGAGGGGAGGAGCCAAAAGATCAGGTAGCTACCTCTGAGAGTGAACTAGGTAAGGCTGTTCTTATGGAACTGTCAGGGCAAGATGTTGAAGCATCACCAGTCGTGGCTGCAGAGGCCGGAGCTGAAGTCTCTGAGAAGCCAGGGCAGGAGATTACAGTTATTCCCAACAATGGTCCAGTTGTTGGACAATCAACTGTAGGAGATCAGACTCCTAGTGAACCACAGACTTCTGCAGAAAGACTGACAGAAACTAAAGATGGCTCAAGTGTAGAGGAGGTCAAGGCAGAGCTGGTTCCTGAACAGGAGGAAGCTATGCTACCTGTAGAAGAGTCTGAGGCAGCTGGAGATGGGGTTGAGACCAAGGTAGCCCAGAGGGCCACGGAGAAAGCACCTGAAGACAAATTTAAGATAGCTGCTAATGAAGAGACACAAGAGAGAGACGAACAGATGAAAGAGGGTGAAG

**Tra2A Poison Exon**

GTTAATGTTCGTGAAGAAATTGAAGAGTTTTTTCCAAGAATGTGGAAGATAAATCAAGATAAAAGAAGGCTAATGAAAAGTATTAAAGATCAGAAAATTAAAATTGAATGGGGGAAAAAATTGAACAGAAGATTGGTCAGATAGAAGCACTTGAATATTTTTTTAAGGCTATTTTGAATTGTTTGAATTGGGGAAGAATACACGAAGTATGAAAAATGAAAAACTCAATGAAGAATGAAGAGAAGTAGAAAGCAAGAGTGAAGTAGAATTAAAAGAATCTGGAAGAATGAATGGGTCCTAGGTTAA

**CREB Exon gamma**

TCATTGTTCTAGGTGCTATCAAAGGCAGCAGTGAACAAACAGTACATCTCTGTTCTCATGAAGATCACATTTGAGTGAAGAGAGACATACTATGAGAAAATAAAATATGTCAG

**FABP9**

AGCCTTATAACCTTTGAAGGTGGGTCAATGATCCAGGTCCAAAAATGGCTTGGCAAACAGACAACAATTAAAAGAAAAATTGTGGATGGAAAAATGGTAGTG

**KRBA1**

GGATGAGACACCTCCAGACTCCTCCCCACCCTAGTCATGAAGCTGGCAGTATGCTTGCCACGGTGAAGGTAGAAGATGGCTGGGCCCAGAGTCCCCCAGTGCCAGCATCCTGCCAGCTTAGCAGGCAAGGCTATAGCTCCTATTCCACTGGAGACAACAGAGAGGTCCGTGTGCCCCGCTGGGGCCCCATGACTCTAG

**Pank2**

ATAGGTGACCTTCAGCTTCGAAAACTGGATGAACTAGATTGCTTAATAAAAGGAATTTTATACATTGACTCAGTTGGATTCAATGGACGGTCACAGTGCTATTATTTTGAAAACCCTGCTGATTCTGAAAAATGTCAGAAGTTACCATTTGATTTAAAAAATCCATATCCTCTGCTTCTGGTGAACATCGGCTCAGGGGTTAGCATCTTAGCAGTGTATTCCAAAGATAATTATAAGAGGGTCACAGGCACCAG

**CREB Exon 2**

GTAACTAAATGACCATGGAATCTGGAGCAGACAACCAGCAGAGTGGAGATGCTGCTGTAACAGAAGCTGAAAATCAACAAATGACAGTTCAAGCCCAGCCACAGATTGCCACATTAGCCCAG

**Lin28b**

GTTCTTCAGAAGATGATGAGGTCATTCAACCAGGGTTCATCAGCCCCAG

**Ankhd1**

GTGCATCCAAGCAGAAGTCCAGTTCTCTGCAGGTAGCAGATCAGGACCTACTGCCACCTTTTCACCCATACCAGCCTTTGGAGTGCATAGTAGAAGAGACTGAAGGCAAGCTGAATGAACTGGGGCAAAGAATTAGTGCTATTGAAAAAGCACAGCTTAAGTCATTGGAGTTAATTCAAGGTGAGCCTCTAAACAAGGATAAGATAGAAGAACTTAAAAAGAACAGAGAAGAGCAAGTCCAGAAGAAGAAGAAAATACTGAAAGAACTACAGAAGGTGGAAAGGCAGTTACAGATGAAAACACAGCAGCAATTTACCAAAGAATACTTGGAAACAAAAGGTCAGAGAGACACAGAGTCTCCACACCAGCAATGTTCTAATAGAGGAGTCTTCATGGCAGGGGAAGAAGATGGCAGTCTCCCACAGGATCACTCTTCAGAATCACCCCAGCTTGATACAGTCTTATTTAAGGATCATGATATTGATGATAAGCAACAGTCTCCACCATCGGCAGAACAAATTGACTTTGTCCCAGTCCAGCCTCTATCATCTCCACAATGTAACTTTTTCAGTGACTTAGGTTCTAATGGGACAAATTCTCTTGTGCTTCAGAAAGTATCAGGTAACCAGCAGATTGTAGGACAGCCTCAGATTGCGATTGCTGGACATGAGCAGGGGCTATTAGTTCAAGAGCCAGATGGACTCATGGTTGCAACTCCAGCCCAGACGCTTACCGACACTCTTGATGACCTGATAGCAG

**Previously Reported Tra2β Targets:**

**Tau Exon 10**

GTGCAGATAATTAATAAGAAGCTGGATCTTAGCAACGTCCAGTCCAAGTG TGGCTCGAAGGATAATATCAAACACGTCCCGGGTGGAGGCAGT

**SMN 2 Exon 7**

GGTTTCAGACAAAATCAAAAAGAAGGAAGGTGCTCACATTCCTTAAATTAAGGA

**Tra2β Exon 2**

GAATCCCGTTCTGCTTCCAGAAGTGGAAGTGCTCACGGATCGGGGAAATCTGCAAGGCATACCCCTGCAAGGTCTCGCTCCAAGGAAGATTCCAGGCGTTCCAGATCAAAGTCCAGGTCCCGATCTGAATCTAG9

**HIPK3-T Exon**

GATAGAAGATATTGAACATTTTTGTATTTGGTGGGGAGAGAGCTCAGAGGGAGGAAGAAATAGAAGATGCAGAAGAGGATGGACTAATTGATGGAGCAGAGTCTTTGAG
