# Supplementary material for: Identification of Evolutionarily Conserved Exons as Regulated Targets for the Splicing Activator Tra2β in Development
Source: PLoS Genet. 2011 Dec 15;7(12):e1002390. doi: 10.1371/journal.pgen.1002390 (PMC3240583; doi:10.1371/journal.pgen.1002390)
Supplement: Figure S2 — Multiple Tra2β binding sites are phylogenetically conserved in Tra2a poison exons and Nasp-T exons. (A) Sequence of the Tra2a poison exon from mouse. (B) Sequence of Nasp-T exon from mouse. Nucleotides in red are conserved in all vertebrates analysed (mouse, frog, rabbit, human, rat, cow, orang-utan, chimp, macaque, marmoset, guinea pig, dog, horse, elephant, opossum, lizard, zebrafinch, tetraodon, stickleback, medaka, chicken). Nucleotides conserved in all mammals are shown in blue. All other nucleotides are shown in black. The Tra2β binding sites predicted from the k-mer analysis are shaded as indicated in Table S1, and the positions of CLIP tags are underlined (note that some of these underlined regions correspond to multiple overlapping CLIP tags which have been joined in this figure). (DOC) [file pgen.1002390.s003.doc]

**Figure S2**

**(A) Tra2a poison exon: 306 nucleotides**

**GTTAATGTTCGTGAAGAAATTGAAGAGTTTTTTCCAAGAATGTGGAAGAT**

**AAATCAAGATAAAAGAAGGCTAATGAAAAGTATTAAAGATCAGAAAATTA**

**AAATTGAATGGGGGAAAAAATTGAACAGAAGATTGGTCAGATAGAAGCAC**

**TTGAATATTTTTTTAAGGCTATTTTGAATTGTTTGAATTGGGGAAGAATA**

**CACGAAGTATGAAAAATGAAAAACTCAATGAAGAATGAAGAGAAGTAGAA**

**AGCAAGAGTGAAGTAGAATTAAAAGAATCTGGAAGAATGAATGGGTCCTA**

**GGTTAA**

**(Nucleotides conserved in all vertebrates shown in red. Other nucleotides in black).**

**(B) NASP exon: 975 nucleotides**

**AGGAAGCAAGGGAAGAGTTGAGAGAACAGGTTTATGACGCCATGGGAGAA**

**AAAGAAGCCAAAAAAGCAGAAGGCAAGTCTCTGACAAAGCCTGAAACTGA**

**TAAAGAACAGGAAAGTGAAGTGGAGAAGGGTGGAAGAGAAGACATGGATA**

**TAAGTGAGCCTGAAGAGAAGCTCCAGGAAACAGTTGAACCGACTTCAAAG**

**CAGTTAACTGAATCCTCTGAAGAGGCAAAAGAAGCAGCAATACCAGGACT**

**GAATGAAGATGAGGTCGCTTCTGGGAAGACAGAGCAGGAATCATTGTGTA**

**CTGAGAAAGGAAAATCAATTTCAGGAGCTTATGTTCAAAATAAAGAATTC**

**AGAGAAACAGTGGAGGAGGGAGAGGAAATAATAAGCTTAGAGAAAAAGCC**

**AAAAGAAACTTCAGAAGATCAGCCTATCAGGGCTGCAGAAAAGCAGGGCA**

**CTTTAATGAAGGTGGTAGAAATAGAAGCTGAAATAGACCCTCAAGTCAAG**

**TCAGCGGATGTGGGAGGGGAGGAGCCAAAAGATCAGGTAGCTACCTCTGA**

**GAGTGAACTAGGTAAGGCTGTTCTTATGGAACTGTCAGGGCAAGATGTTG**

**AAGCATCACCAGTCGTGGCTGCAGAGGCCGGAGCTGAAGTCTCTGAGAAG**

**CCAGGGCAGGAGATTACAGTTATTCCCAACAATGGTCCAGTTGTTGGACA**

**ATCAACTGTAGGAGATCAGACTCCTAGTGAACCACAGACTTCTGCAGAAA**

**GACTGACAGAAACTAAAGATGGCTCAAGTGTAGAGGAGGTCAAGGCAGAG**

**CTGGTTCCTGAACAGGAGGAAGCTATGCTACCTGTAGAAGAGTCTGAGGC**

**AGCTGGAGATGGGGTTGAGACCAAGGTAGCCCAGAGGGCCACGGAGAAAG**

**CACCTGAAGACAAATTTAAGATAGCTGCTAATGAAGAGACACAAGAGAGA**

**GACGAACAGATGAAAGAGGGTGAAG**

**Nucleotides conserved in all vertebrates shown in red. Nucleotides conserved in placental mammals shown in blue (within some shaded boxes, these are annotated lighter blue to distinguish these nucleotides against the darker high frequency green background. Other nucleotides in black).**

**Key to k-mer annotation in parts (A) and (B):**

NNN: k-mers 1-5

NNN: k-mers 6-10

NNN: k-mers 11-15

NNN: k-mers 16-20

NNN: K-mers 21-30
