# Supplementary figures and images for: Identification of Evolutionarily Conserved Exons as Regulated Targets for the Splicing Activator Tra2β in Development
Source: PLoS Genet. 2011 Dec 15;7(12):e1002390. doi: 10.1371/journal.pgen.1002390 (PMC3240583; doi:10.1371/journal.pgen.1002390)

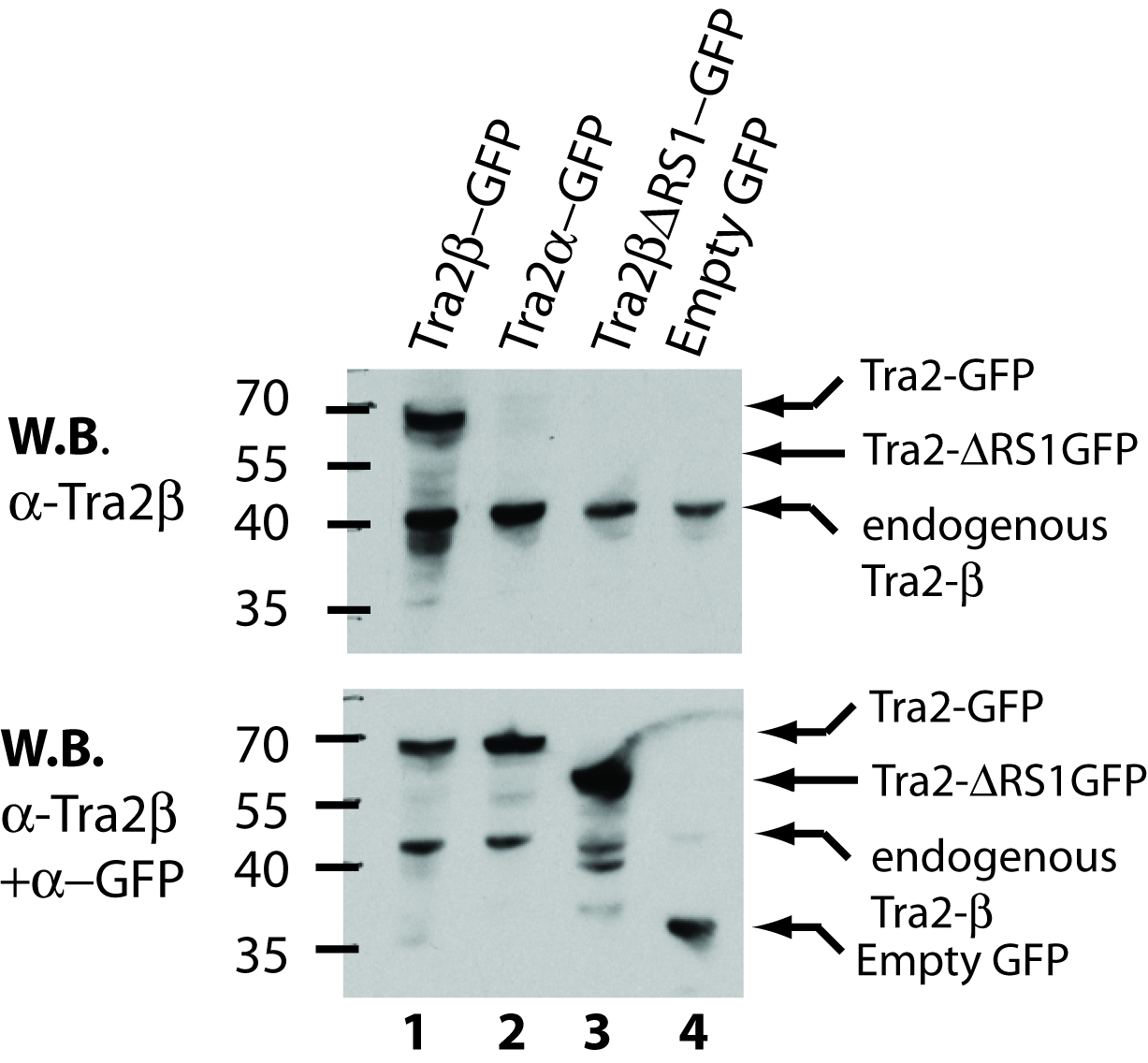

Supplement: Figure S3 — Experiment to confirm the specificity of the polyclonal antisera used for CLIP analysis. HEK293 cells were transfected with plasmids expressing the indicated proteins, proteins isolated and analysed by SDS-PAGE and Western blotting. The same blot was probed sequentially with an affinity purified antisera raised against Tra2β [65] and then with a polyclonal specific for GFP to detect expression of the fusion proteins. The affinity purified α-Tra2β antisera detected a single band in HEK293 cells corresponding to endogenous Tra2β protein, and also the Tra2β-GFP fusion protein. No recognition of either Tra2α or Tra2βΔRS1-GFP was observed, indicating that this antisera is highly specific. (TIF) [file pgen.1002390.s004.tif]
