# Supplementary material for: Identification of Evolutionarily Conserved Exons as Regulated Targets for the Splicing Activator Tra2β in Development
Source: PLoS Genet. 2011 Dec 15;7(12):e1002390. doi: 10.1371/journal.pgen.1002390 (PMC3240583; doi:10.1371/journal.pgen.1002390)
Supplement: Table S1 — Properties of the 30 most frequently retrieved 6-mers in the Tra2β CLIP tags. The 6-mers are ordered from the most frequently recovered at the top of the table (AGAAGA) to the 34th most frequently recovered 6-mer at the bottom (GAAGCT). The 6-mers are arranged in colour blocks of 5 according to their frequency of retrieval, and compared and corrected with their frequencies in both the total mouse genome and mouse testis transcriptome. The same colour code of the different 6-mer categories are also used to illustrate the occurrence of these 6-mers within the Tra2β target exons in Figures S1 and S2. (DOC) [file pgen.1002390.s005.doc]

**Table S**1

| **6-mer** | **Frequency of 6-mer in CLIP tags (S)** | **Frequency of 6-mer in genome** | **Genome corrected frequency (G)** | **Ranking (G)** | **Frequency of 6-mer in mouse testis tran-scriptome** | **Transc-riptome corrected frequency (T)** | **Ranking (T)** |
| --- | --- | --- | --- | --- | --- | --- | --- |
| AGAAGA | 5.61687 | 0.770722 | 4.84615 | 1 | 1.15402 | 4.46285 | 1 |
| GAAGAA | 4.86716 | 0.671663 | 4.1955 | 2 | 0.854474 | 4.01269 | 2 |
| AAGAAG | 4.29174 | 0.660342 | 3.6314 | 3 | 0.878069 | 3.41367 | 3 |
| TGAAGA | 3.95867 | 0.516819 | 3.44185 | 4 | 0.991471 | 2.9672 | 4 |
| GAAGAT | 3.42623 | 0.371741 | 3.05449 | 5 | 0.60352 | 2.82271 | 5 |
| AAGAAA | 3.55635 | 1.16015 | 2.3962 | 6 | 1.05244 | 2.50391 | 6 |
| GAAGAG | 2.64727 | 0.491786 | 2.15548 | 7 | 0.719154 | 1.92812 | 8 |
| GGAAGA | 2.62936 | 0.50618 | 2.12318 | 8 | 0.721032 | 1.90833 | 9 |
| CTGAAG | 2.24496 | 0.439994 | 1.80497 | 9 | 0.732098 | 1.51286 | 11 |
| GAGAAG | 2.24854 | 0.480449 | 1.76809 | 10 | 0.732883 | 1.51566 | 10 |
| AAAGAA | 2.8729 | 1.15234 | 1.72056 | 11 | 0.888027 | 1.98487 | 7 |
| AAGATG | 2.05633 | 0.4243 | 1.63203 | 12 | 0.693867 | 1.36246 | 14 |
| CAGAAG | 2.18049 | 0.562355 | 1.61813 | 13 | 0.954301 | 1.22619 | 16 |
| GAAGAC | 1.89636 | 0.307239 | 1.58912 | 14 | 0.77801 | 1.11835 | 20 |
| ATGAAG | 1.92024 | 0.4163 | 1.50394 | 15 | 0.556977 | 1.36326 | 13 |
| AGAAAA | 2.43835 | 1.04237 | 1.39598 | 16 | 0.943035 | 1.49531 | 12 |
| AGGAAG | 2.01515 | 0.686074 | 1.32908 | 17 | 0.745566 | 1.26958 | 15 |
| AAGAGA | 1.95844 | 0.634115 | 1.32432 | 18 | 0.771916 | 1.18652 | 18 |
| AGAGAA | 2.07364 | 0.77395 | 1.29969 | 19 | 0.858814 | 1.21483 | 17 |
| CAAGAA | 1.7167 | 0.471291 | 1.24541 | 20 | 0.567135 | 1.14956 | 19 |
| AGAAGC | 1.62119 | 0.399502 | 1.22169 | 21 | 0.639289 | 0.981901 | 24 |
| TTGAAG | 1.44033 | 0.372888 | 1.06744 | 22 | 0.529042 | 0.911288 | 25 |
| AGAAAG | 1.87965 | 0.85311 | 1.02654 | 23 | 0.884595 | 0.995055 | 22 |
| GTGAAG | 1.23798 | 0.26664 | 0.97134 | 24 | 0.398709 | 0.839271 | 30 |
| AAGAAC | 1.36691 | 0.408793 | 0.958117 | 25 | 0.378516 | 0.988394 | 23 |
| GAAAGA | 1.68506 | 0.728353 | 0.956707 | 26 | 0.685001 | 1.00006 | 21 |
| AGAAGT | 1.33468 | 0.394921 | 0.939759 | 27 | 0.577817 | 0.756863 | 36 |
| AGATGA | 1.33169 | 0.393388 | 0.938302 | 28 | 0.630116 | 0.701574 | 41 |
| GATGAA | 1.23201 | 0.313878 | 0.918132 | 29 | 0.453148 | 0.778862 | 33 |
| GAAGCT | 1.23261 | 0.319311 | 0.913299 | 30 | 0.454841 | 0.777769 | 34 |
