# Supplementary material for: Identification of Evolutionarily Conserved Exons as Regulated Targets for the Splicing Activator Tra2β in Development
Source: PLoS Genet. 2011 Dec 15;7(12):e1002390. doi: 10.1371/journal.pgen.1002390 (PMC3240583; doi:10.1371/journal.pgen.1002390)
Supplement: Table S3 — Top functions associated with Tra2β-bound mRNAs determined from Ingenuity Pathway Analysis (IPA). (DOCX) [file pgen.1002390.s007.docx]

| **Category** | **Name** | **p-value** | **Number of molecules** |
| --- | --- | --- | --- |
| **Diseases and Disorders** | Genetic Disorder | 2.12E-65 - 2.30E-03 | 3502 |
|  | Neurological Disease | 5.13E-50 - 1.86E-03 | 2111 |
|  | Gastrointestinal Disease | 8.43E-40 - 3.08E-04 | 1819 |
|  | Metabolic Disease | 8.70E-40 - 2.25E-19 | 1519 |
|  | Endocrine System Disorders | 6.32E-39 - 2.25E-19 | 1365 |
| **Molecular and Cellular Functions** | Post-Translational Modification | 3.22E-38 - 1.73E-03 | 736 |
|  | Cell Cycle | 1.31E-34 - 2.28E-03 | 808 |
|  | Gene Expression | 9.52E-21 - 2.10E-03 | 1015 |
|  | RNA Post-Transcriptional Modification | 6.89E-18 - 5.75E-06 | 192 |
|  | Cell Death | 5.60E-17 - 2.14E-03 | 1400 |
| **Physiological System Development and Function** | Reproductive System Development and Function | 4.57E-14 - 2.03E-03 | 241 |
|  | Organismal Development | 2.98E-13 - 1.46E-03 | 596 |
|  | Organismal Survival | 4.45E-07 - 9.66E-07 | 470 |
|  | Tumor Morphology | 1.44E-06 - 8.57E-04 | 114 |
|  | Nervous System Development and Function | 4.15E-06 - 2.30E-03 | 305 |
| **Top canonical pathways** | Inositol Phosphate Metabolism | 2.81E-15 | Ratio 95/138  (0.688) |
|  | Protein Ubiquitination Pathway | 4.29E-14 | Ratio 155/270  (0.574) |
|  | Protein Kinase A Signaling | 1.14E-11 | Ratio 170/315  (0.54) |
|  | ERK/MAPK Signaling | 7.8E-10 | Ratio 108/198  (0.545) |
|  | FAK Signaling | 8.22E-10 | Ratio 60/99  (0.606) |
